# Hepatotoxicity Comparison of Crude and Licorice-Processed Euodiae Fructus in Rats With Stomach Excess-Cold Syndrome

**DOI:** 10.3389/fphar.2021.756276

**Published:** 2021-11-23

**Authors:** Min Zhang, Meng Gao, Shanshan Wu, Lifen Zhou, Lan Cao, Rifa Qiao, Minyong Zhong, Lin Yang, Jinbin Yuan

**Affiliations:** ^1^ Key Laboratory of Modern Preparation of TCM, Ministry of Education, Jiangxi University of Chinese Medicine, Nanchang, China; ^2^ Nanchang Key Laboratory of Detection and Control of Food Safety, Nanchang Inspection and Testing Center, Nanchang, China; ^3^ Research Center for Traditional Chinese Medicine Resources and Ethnic Minority Medicine, Jiangxi University of Chinese Medicine, Nanchang, China

**Keywords:** *Tetradium ruticarpum* (A. Juss.) Hartley, euodiae fructus, hepatotoxicity, drug processing, licorice processing, detoxification, stomach excess-cold syndrome

## Abstract

In recent years, drug-induced liver injury (DILI) has become an important issue of public health. Euodiae Fructus (EF) is a commonly used herb with mild toxicity in clinic, and large doses of EF can cause significant liver damage. Licorice processing might reduce the hepatotoxicity of CEF (crude EF), but up to now, studies on the hepatotoxicity of EF have been hardly reported, let alone its material basis and mechanism of detoxification by licorice processing. This work firstly established a stomach excess-cold syndrome animal model induced by intragastric administration of cold *Zhimu* (*Anemarrhena asphodeloides* Bge). Secondly, multiple approaches and indexes were used to evaluate the hepatotoxicity of the drugs in the rats including general behavior, biochemical analysis, protein expressions, and histopathological examination. Thirdly, the hepatotoxicity of three doses of three CEF and LPEF (licorice-processed EF) extracts was systematically investigated, and the hepatotoxicity differences were analyzed and compared comprehensively among the three extracts, three doses, and CEF and LPEF. Finally, the connotation of detoxification of EF by licorice processing was preliminarily discussed according to the changes in toxic components after processing, toxicological characteristics, and TCM (traditional Chinese medicine) theory. All extracts of EF were found to have dose-dependent hepatotoxicity, and the toxicity was in the descending order of water extract, ethanol extract, and volatile oil. The hepatotoxic mechanism of EF may be related to peroxidation damage, inflammatory factor, and mitochondrial injury. The CEF hepatotoxicity can be significantly reduced by licorice processing. EF should be safe for short-term use at pharmacopeial dose under the guidance of the TCM theory. The detoxification mechanism is probably related to the reduction of toxic components and antagonistic action of licorice.

## Introduction

Throughout human history, traditional medicine has made important contributions to the prevention and treatment of the diseases all over the world. Safety has always been the outstanding advantage of traditional Chinese medicine (TCM). With the widespread use of TCM in the world, however, the safety problems/issues are gradually increasing (Teschke and Eickhoff, 2015; Xiao et al., 2021). Especially in recent years, the frequent occurrence of adverse events, such as drug-induced liver injury (DILI), has become an important issue for public health. The TCM safety has become a domestic and international focus, which has seriously affected the healthy and sustainable development as well as the modernization and internationalization process of TCM (Wang et al., 2018; Xiao, 2019). The safe use of herbal medicine and traditional medicine has been increasingly challenging. Therefore, it is of great importance to study the toxic and side effects of TCM and explore the scientific connotation of detoxification.

Euodiae Fructus (EF) is the dry, nearly ripe fruit of *Tetradium ruticarpum* (A. Juss.) Hartley (Shan et al., 2020). EF has been long used in China, being famous for the remarkable function of “warming Middle-jiao to dispel cold” in TCM theory, and it is chiefly used in treating stomach excess-cold syndrome (Qin et al., 2013). Modern pharmacological studies have confirmed that EF has analgesic, anti-inflammatory, anti-ulcer, anti-tumor, and other effects. Crude Euodiae Fructus (CEF) has been described as “mildly toxic” in classic medical books since ancient times (for example, Shennong Materia Medica in Han dynasty). However, the traditional knowledge of TCM is a little indistinct about toxicity cognition and intervention countermeasures, and it is necessary to carry out systematic modern toxicological studies on Euodia Fructus. Earlier, Yang et al. (2008) paid attention to the toxic and side effects of EF and studied the acute toxicity and genetic toxicity of its water extract and 70% ethanol extract, but no significant toxicity was found. Since then, toxicity studies of EF have been growing year by year, and it has been found that the volatile oil (VO) (Sun et al., 2012; Yin et al., 2015), ethanol extract (EE) (Liu et al., 2015; Liang et al., 2017), and water extract (WE) (Zhou et al., 2011; Huang et al., 2012; Huang and Sun., 2012; Zhou et al., 2013; Cai et al., 2014) of EF have definite hepatotoxicity. Clinical toxicity cases are often caused by oral administration of unprocessed EF or overdose accompanied by abnormal liver function (Cohen et al., 2012). However, it has not been confirmed whether there are differences regarding hepatotoxicity among different fractions of EF and, if there were, which fraction or components are more toxic.

Drug processing is a traditional pharmaceutical technique in China and plays an important role in reducing the toxicity of crude drug. To reduce the toxic and side effects of EF, many processing methods have been developed, such as washing with hot or cold water, stir-frying with ginger juice, vinegar, wine, licorice, and *Coptidis Rhizoma* water extract (Xiao et al., 2017; Li et al., 2020; Shan et al., 2020). Licorice processing is the most common one, which may be due to the fact that licorice is good at moderating toxic herbs (Gong et al., 2015). Licorice-processed Euodiae fructus (LPEF) was recorded in the current Chinese Pharmacopoeia (Commission of Chinese Pharmacopoeia, 2020) as the typical processed product of EF. There have been some reports about reducing EF toxicity by drug processing; however, the studies of reducing EF hepatotoxicity by licorice processing were mainly the work of Zhong Zhenguo’s group (Zhang et al., 2017; Liu et al., 2018; Zhang et al., 2018). The group successively compared the hepatotoxicity of different processed products through the *in vitro* toxicity test of L-02 cells (Zhang et al., 2017) and *in vivo* hepatotoxicity test of mice (Liu et al., 2018), and found that the hepatotoxicity of LPEF was significantly lower than that of CEF (Zhang et al., 2018). It should be pointed out that the main purpose of Zhong’s work (Zhang et al., 2017; Liu et al., 2018; Zhang et al., 2018) was to roughly compare the toxicity of the water extract of various processed products (the raw product, the licorice-processed product, and the salt-processed product) with limited experiments and detection indexes. Therefore, a more comprehensive comparison of hepatotoxicity between CEF and LPEF and the detoxification mechanism by drug processing still need to be further investigated.

Modern pharmacological studies show that a drug often has some side effects while exerting its therapeutic effects, and some significant differences in efficacy/toxicity are often observed between normal and model animals, or among different models. Therefore, the toxicity of a drug should be reasonably evaluated and scientifically recognized under the background of syndrome. However, most of the above literatures on hepatotoxicity are based on normal animals, and the relevant results may not reflect the exact toxicity under the actual pathological condition. More systematic and comprehensive hepatotoxicity data of CEF and LPEF need to be further studied based on the corresponding syndrome.

In this work, we firstly established a stomach excess-cold syndrome animal model. Then, the hepatotoxicity of three doses of three CEF and LPEF extracts (WE, EE, and VO) was systematically investigated according to the general behavior, biochemical analysis, protein expression and histopathological examination. The differences in hepatotoxicity were analyzed and compared comprehensively among different extracts, three doses, CEF, and LPEF. Finally, the connotation of detoxification of EF processed with licorice was preliminarily discussed according to the changes of toxic components after processed, toxicological characteristics, and TCM theory.

## Materials and Methods

### Crude Drugs

EF was collected from Shuangjin GAP (good agriculture practice) Planting Base, Zhangshu, Jiangxi province, China in August 2020. All the collected samples were identified as the nearly ripe fruit of *Tetradium ruticarpum* (A. Juss.) Hartley by Professor Lan Cao of Jiangxi University of Chinese Medicine (JXUCM). *Glycyrrhiza uralensis* Fisch*.* (licorice) (Batch No. 20200514) and *Anemarrhena asphodeloides* Bge (“*Zhimu*” in Chinese) (Batch No. 20200905) were bought from Jiangxi Huangqingrenzhan Huashi Pharmacy Co., Ltd. (Nanchang, China), and were identified by Professor Lan Cao, too. LPEF was processed with licorice according to the Chinese Pharmacopoeia (Commission of Chinese Pharmacopoeia, 2020). The plant materials were dried in the shade and stored in a dry and cool place until use. Voucher specimens are preserved in the Herbarium of Pharmacognosy in JXUCM, and the numbers of specimens are in the order of ZS20200815001, GC20200514001, and ZM20200905001, respectively.

### Licorice-Processed Euodiae Fructus

LPEF is the licorice-processed product of CEF and recorded in Chinese Pharmacopoeia. The technology of licorice processing strictly followed the pharmacopoeia (Commission of Chinese Pharmacopoeia, 2020), and was outlined as follows: ① licorice was crushed and soaked with water for 2 h, and decocted for 2 h to obtain licorice juice; ② EF was weighed, and then soaked with licorice juice (100 g of EF vs. 600 g of licorice); the mixture was then stir-fried (at 120°C) until it was slightly dry; and ③ the processed EF was further dried in a drying oven at 105°C for 2 h; then, it was weighed and LPEF was obtained.

### Chemicals and Reagents

SOD kit (batch No. 20201125), MDA kit (batch No. 20201125), rat IL-6 ELSA Kit (batch No. 20201127), rat IL-1*β* ELSA Kit (batch No. 20201127), rat TNF-*α* kit (batch No. 20201127), ATP enzymes kit (batch No. 20201125), GOT kit (batch No. 20201121), and GPT kit (batch No. 20201121) were obtained from Nanjing Jiancheng Bioengineering Institute (Nanjing, China). BCA protein quantitative kit (No. PC0020) was purchased from Beijing Solarbio Science & Technology Co., Ltd. (Beijing, China). GAPDH (No. vab181602), Bcl-2 (No. ab59348), Bax (No. ab32503), and Caspase-3 (No. ab2302) were from Abcam, Cambridge, United Kingdom HRP-conjugated goat anti-rabbit IgG (No. S004F) was from Beijing TDY Biotech CO., Ltd. (Beijing, China). Acetaminophen was provided by Hebei Jiheng Pharmaceutical Co., Ltd. (Hengshui, Hebei, China).

Nine standards were purchased from National Institute for the Control of Pharmaceutical and Biological Products (Beijing, China), including chlorogenic acid (No. 110753–201817), rutin (No. 100080–201811), hyperoside (No. 111521–201406), liquiritin (No. 111610–201607), quercetin (No. 100081–201610), glycyrrhizic acid (No. 110731–201720), evodiamine (No. 110802–201710), rutaecarpine (No. 110801–201608), and glycyrrhetinic acid (No. 110723–200310). Eight standards were from Chengdu Push Biotech Co., Ltd. (Chengdu, China), namely, neochlorogenic acid (No. PS000974), cryptochlorogenic acid (No. PS001110), caffeic acid (No. PS010522), isorhamnetin-3-O-rutinoside (No. PS010525), dehydroevodiamine (No. PS200709-01), limonin (No. PS010690), evocarpine (No. PS200709-02), and dihydroevocarpine (No. PS200709-03). The nominal contents of the above standards are more than 95.0% with HPLC (high-performance liquid chromatography) detection.

All the other chemicals and solvents were of analytical grade.

### Animals and Establishment of the Stomach Excess-Cold Syndrome Model

Adult male Sprague-Dawley (SD) rats [Certificate No. SCXK (Xiang) 2019-0004] weighing 180 ± 20 g were purchased from Hunan Silaike Laboratory Animal Ltd. (Changsha, China). The animals were maintained in a controlled breeding room under the following conditions: temperature (22 ± 2°C), relative humidity (65 ± 5)%, 12-h light–dark cycles (Yuan et al., 2017). The Experimental Animal Ethic Committee of JXUCM approved all animal protocols (Certificate No. JZLLSC20210017). The animal experiments were carried out according to the European Community Guidelines for the Use of Experimental Animals.

All rats except the control group (10 rats) were fed with the decoction of 0.5 g kg^−1^ 4°C *Zhimu* at a dose of 20 ml kg^−1^, twice a day for 2 days to establish the stomach excess-cold model (Qin et al., 2013).

### Sample Preparation

*Zhimu* decoction: The sample preparation procedure was similar to the literature (Qin et al., 2013). A certain amount of *Zhimu* was crushed, and it was then added with 10 times water. After soaking for 2 h, the sample was decocted for 2 h to obtain the filtrate. The residue was added with eight times water and decocted for another 1 h, and then filtered. The two filtrates were combined and concentrated in a rotary evaporator (N-1300, Tokyo Rikakikai Co., Ltd., Shanghai, China) to 0.5 g crude drug per milliliter. The decoction was stored at 4°C for later use.

Water extract (WE): The sample preparation procedure was modified according to our previous experiments (Dong, 2018; Li et al., 2019). A certain amount of CEF powders (2,500 g) were added with 10 times water. After soaking for 2 h, the samples were decocted for 2 h to obtain the filtrates. The residues were added with eight times water and decocted for another 1 h, and then filtered. The two filtrates were combined and concentrated in a rotary evaporator (N-1300) to 2 g crude drug per milliliter. The extracts were stored at 4°C till further analysis. The water extract of LPEF was prepared with the same method as above.

Ethanol extract (EE): The sample preparation was similar to the above procedures, except that the solvent was replaced by 70% ethanol.

Volatile oil (VO): VO was extracted by steam distillation, and its volume was determined by method A according to Chinese Pharmacopoeia (Commission of Chinese Pharmacopoeia, 2020). CEF or LPEF (300 g) was placed in a 5,000-ml flask and soaked for 2 h with 10 times water. After extracting for 8 h, the collected oil was left to rest for an hour, and the volume of volatile oil was read. Fifty microliters of volatile oil was sampled for GC-MS (gas chromatography-mass spectrometry) analysis (Li et al., 2017). The rest of the volatile oil was dissolved in Tween-80 at a ratio of 1:1 and stored at 4°C for animal experiments.

### Analysis of the Extracts

The UHPLC (ultra-high performance liquid chromatography) analysis of WE and EE was performed according to our previous work (Dong et al., 2018; Li et al., 2019). The UHPLC system was Agilent 1260 system (Agilent Technologies, Inc., United States), which consisted of a DGU-20A5R degasser, a G7129A vial sampler, a G7115A DAD WR, a G7116A MCT, and a G7111A Quat pump VL. The separation was carried out on an Agilent C18 column (2.1 × 100 mm, 1.8 mm, Agilent Technologies, Inc., United States) with temperature at 40°C. The mobile phase was composed of water (containing 0.1% formic acid, solvent A) and Acetonitrile (solvent B). The gradient elution procedure was (0.01–5 min, 30–50% B, 5–7 min, 50% B, 7–7.5 min, 50–30% B, 7.5–10.5 min, 30% B). The flow rate was 0.3 ml/min and injection volume was 5.0 μl.

The volatile oil samples were subjected to GC-MS analysis using an Agilent 7890A-5975c and a DB-17MS column (length 30 m, inner diameter 0.25 mm, film thickness 0.25 μm) according to the modified method (Li et al., 2017).

### Groups, Doses, and the Procedures of Hepatotoxicity Experiment

After the model was established, 200 model rats were divided into groups with 10 rats for each group. Groups and dose information are shown in Table 1. The rats were given a dose of drug (10 ml kg^−1^) to all groups by gavage once per day for 15 days consecutively, and the same dose of normal saline was given to the control group and the model group.

**TABLE 1:** Groups and doses of experimental animals.

Group		Dose
Name	Symbol	
Control	Control	—
Acetaminophen	APAP	0.21 mg kg^−1^
Model	Model	—
Crude Euodiae Fructus	CEF	—
Licorice-processed Euodiae Fructus	LPEF	Low dose (L): 1.05 g kg^−1^
Water extract	WE	Medium dose (M): 5.25 g kg^−1^
70% ethanol extract	EE	High dose (H): 10.5 g kg^−1^
Volatile oil	VO	—

The animal’s general behavior including diet, drinking, mental state, hair, weight, urine, and feces was recorded throughout the 15 days. The general symptoms of toxicity were also monitored once a day. All rats were weighed every 5 days during the period of treatment, and the changes in body weight were recorded and calculated. On the 15th day, all rats were fasted overnight and sacrificed afterwards for blood collection from abdominal aorta. Immediately after collecting the blood samples, the livers were removed and weighed. The relative liver/body weight ratio of each rat was calculated. The livers were then used for Western blotting, ATP enzyme analysis, and pathological investigation.

### Determination of ALT, AST, IL-6, IL-1*β*, TNF-*α*, SOD, MDA, and Mitochondrial ATP Enzymes

After the last administration on the 15th day, the rats were sacrificed and their blood was collected into EP tubes, blood samples were centrifuged at 4,500 rpm for 10 min, the supernatant was separated, and the serum was frozen and stored at −80°C before analysis; livers were separated and washed with saline and weighed, and the ratio of liver weight to terminal body weight were calculated. Frozen livers were weighed and crumbled by surgical scissors at icy temperature and accurately weighed. Liver samples (1 g) and 9 ml of precooled saline were mixed with a tissue homogenizer at 10,000 rpm to prepare a 10% homogenate. The 10% liver homogenate samples were then centrifuged for 10 min, and the supernatant was removed and stored at −80°C. Levels of ALT, AST, IL-6, IL-1*β*, TNF-*α*, SOD, and MDA in the blood, and Na^+^-K^+^-ATPase and Ca2^+^-Mg2^+^-ATPase in livers were measured according to the kit instructions.

### Western Blotting Analyses of Bcl-2, Bax, and Caspase-3 Protein

Frozen livers were weighed and crumbled by surgical scissors at icy temperature and homogenized in lysis buffer (50 mmol L^−1^ Tris, 0.25% sodium deoxycholate, 1% NP-40, 1 mmol L^−1^ EDTA, 1 mmol L^−1^ PMSF, 1 mg ml^−1^ aprotinin, and 1 mg ml^−1^ leupeptin) and then were centrifuged for 10 min at 13,000 rpm and 4°C for supernatant. Following determination of the protein concentration by BCA protein quantitative kit, the remainder (30 mg of protein) was subjected to SDS-polyacrylamide gel electrophoresis (PAGE). Separated proteins were transferred to a polyvinylidene difluoride filter (PVDF) membrane *via* transfer apparatus at 72 v for 120 min. The membranes were then blocked *via* 5% bovine serum albumin and then incubated with primary antibody against GAPDH (No. ab181602, Abcam, Cambridge, United Kingdom), Bcl-2 (No. ab59348, Abcam, Cambridge, United Kingdom), Bax (No. ab32503, Abcam, Cambridge, United Kingdom), and Caspase-3 (No. ab2302, Abcam, Cambridge, United Kingdom). Membranes were washed three times (15, 5, and 5 min, respectively) with 5% milk/PBS and incubated with secondary antibody (HRP-conjugated goat anti-rabbit IgG, 0.04 mg ml^−1^, No. S004F, Beijing TDY Biotech Co., Ltd., Beijing, China) for 2 h. Then, after washing with PBS for three times (15, 5, and 5 min, respectively), the membranes were visualized by ChemiScope Mini 3300 (Clinx Science Instruments Co., Ltd., Shanghai, China) and scanned with ImageJ software. For negative controls, primary antibodies were replaced with normal IgG at a similar concentration and origin.

### Histopathological Examination

The collected gastric mucosa and livers were kept in 10% neutral buffered formalin for 48 h and then embedded within paraffin. Sections of 4 µm thickness were prepared using a rotary microtome. The sections were stained with hematoxylin and eosin (H and E). The morphological characteristics were assessed by an electron microscope (Leica Microsystems, DM1000, Wetzlar, Germany).

### Statistical Analysis

All statistical analyses were performed by SPSS 21.0 software package from SPSS, Inc. (Chicago, IL, United States). One-way ANOVA analysis was used for the comparisons among different groups (*n* = 10). * *p* < 0.05 and ** *p* < 0.01 are considered statistically significant difference compared to the control group; ^#^ *p* < 0.05 and ^##^ *p* < 0.01 are considered as statistically significant difference compared to the unprocessed group; ^◇^ *p* < 0.05 and ^◇◇^ *p* < 0.01 are considered as statistically significant difference compared to the APAP group.

## Results

### Confirmation of the Stomach Excess-Cold Syndrome Model

**FIGURE 1:**
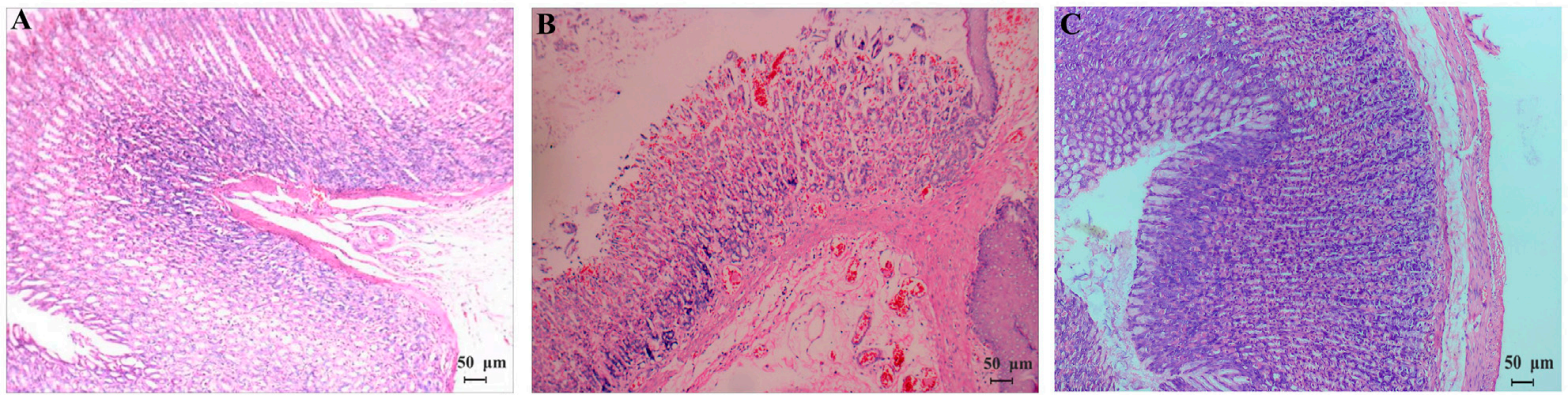
Pathomorphology (HE, ×100) of gastric mucosa of normal rats **(A)** and model rats [**(B)** the 2nd day; **(C)** the 15th day]. **(A,C)** Cells are uniform in size and evenly distributed with clear boundaries; **(B)** gastric mucosa had inflammatory cells infiltrated and gland destruction, hyperemia and edema, and blurry cell boundaries.

The animal model was evaluated according to the general behavior and the pathological examination of the model rats (Huang et al., 2014). During the 2-day model establishment, the rat feces in the model group were heavy, wet and soft, even unformed, their hair was dirty and messy, their mental state was poor, and the animals were reluctant to move and had low appetite. More data on the general behavior and gastrointestinal functions of the rats after modeling are summarized in Table 2, and the significant differences can be observed between the control and model groups. As shown in Figure 1, the gastric mucosa of model rats showed increased neutrophil count, gland destruction, hyperemia, edema, and blurry cell boundary (Figure 1B). By the end of the 15th day, the gastric mucosa of the model group was basically restored back to the normal morphology (Figure 1C). The above symptoms and pathology confirmed that the model was successfully established.

**TABLE 2:** Comparison of general behaviors and gastrointestinal functions of the rats after modeling (mean ± SD, *n* = 10).

Group	Body weight (g)		Gastric acidity^a^ (mmol·L^−1^)	Food intake (g·day^−1^)	Water intake (ml·day^−1^)	Feces (0–12 h)		Urine (0–12 h)	
	0 days	2nd day				Weight (g)	Feature	Volume(ml)	Color
Control	183.5 ± 6.9	193.2 ± 11.5	27.3 ± 2.8	22.4 ± 2.3	25.7 ± 2.3	8.2 ± 0.5	Dry, granular	13.6 ± 0.8	Faint yellow
Model	181.1 ± 8.8	180.4 ± 7.4*	45.6 ± 5.9**	15.4 ± 1.2**	15.8 ± 1.3**	12.6 ± 1.3**	Wet, unformed	9.4 ± 0.5**	Dark yellow

a At 48 h, *n* = 5.

Vs. control group, *p* < 0.05 (*), *p* < 0.01 (**).

### Changes of the Components Before and After Processing

**FIGURE 2:**
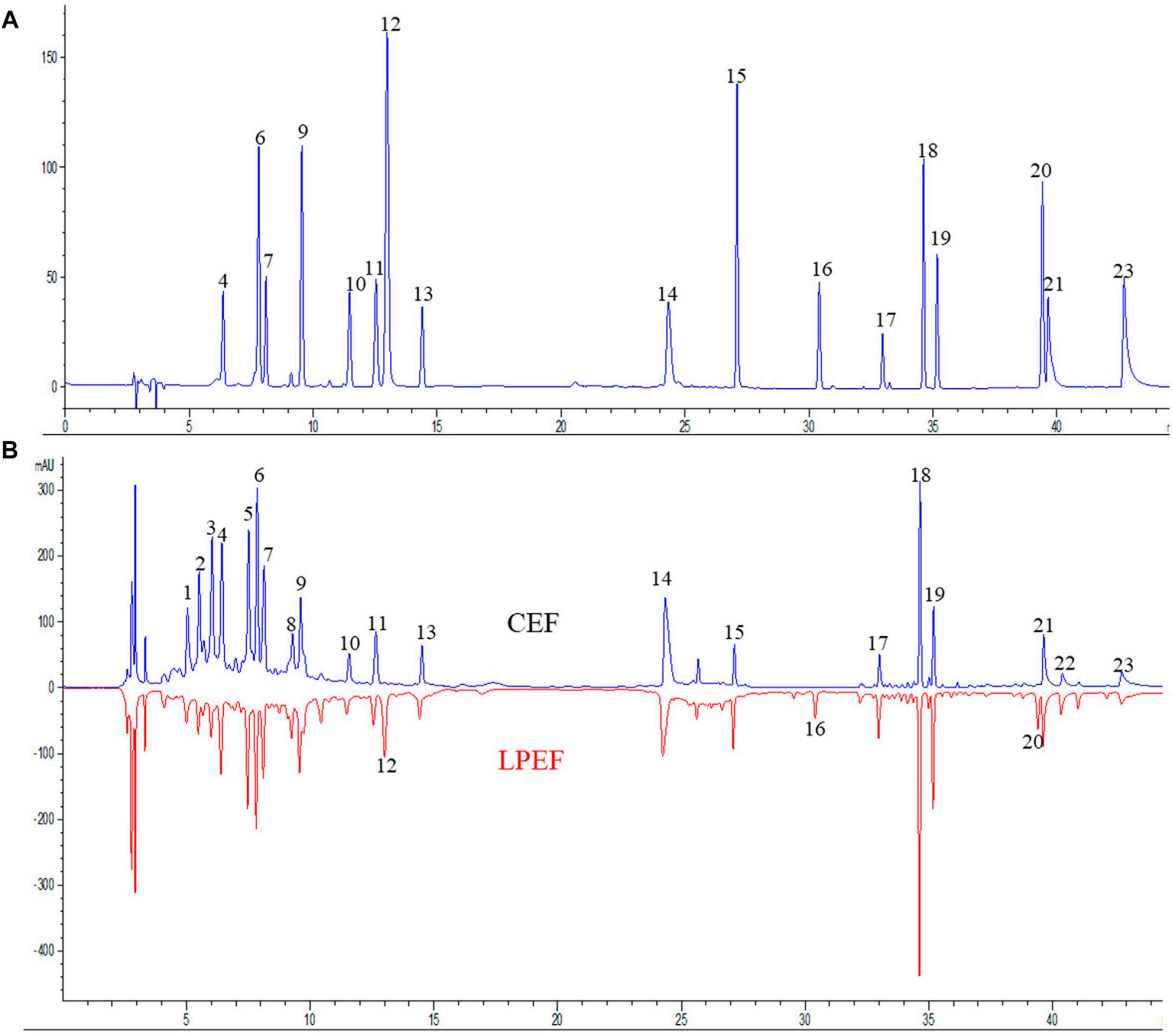
Typical UHPLC chromatograms [**(A)** chromatogram of 17 mixed standards, **(B)** fingerprints of CEF and LPEF]. Identification of peaks is shown in Table 3.

The color of LPEF is significantly darker than that of CEF, but their weight is almost unchanged in this work. To keep track of the compositional changes of EF after processing, an integrated method of fingerprint qualitative and multi-component quantitative analysis was developed and validated based on our early work (Dong et al., 2018; Li et al., 2019). The compounds were identified using UHPLC-Q-TOF/MS (ultra-high-performance liquid chromatography coupled with quadrupole-time-of-flight mass spectrometry) technique according to our previous research (Liang et al., 2017; Dong, 2019; Li, 2019). Relevant MS information is presented in Supplementary Table S1. More organic acids (mainly including chlorogenic acids and caffeoyl gluconic acids) and flavonoid glycosides were found in WE and more indoles and quinolone alkaloids in EE. The typical chromatograms are shown in Figure 2, and the contents of main components in CEF and LPEF are summarized in Table 3. The main chemical changes were a significant reduction in some organic acids and the presence of licorice components such as liquiritin and glycyrrhizic acid after licorice processing.

**TABLE 3:** Common peaks and their contents in LPEF and CEF (mean ± SD, *n* = 6).

Peak No.	Compound	In CEF (%)	In LPEF (%)
1	Trans-caffeoylgluconic acid-d1	/	/
2	Trans-caffeoylgluconic acid-d2	/	/
3	Trans-caffeoylgluconic acid-d3	/	/
4	Neochlorogenic acid	1.276 ± 0.056	0.536 ± 0.022
5	Trans-caffeoylgluconic acid-d4	/	/
6	Chlorogenic acid	0.452 ± 0.017	0.314 ± 0.014
7	Cryptochlorogenic acid	1.021 ± 0.044	0.549 ± 0.024
8	Trans-caffeoyl gluconate-methyl ester	/	/
9	Caffeic acid	0.166 ± 0.007	0.340 ± 0.015
10	Rutin	0.038 ± 0.002	0.018 ± 0.001
11	Hyperoside	0.218 ± 0.009	0.109 ± 0.004
12	Liquiritin	/	0.004 ± 0.001
13	Isorhamnetin-3-O-rutinoside	0.146 ± 0.005	0.193 ± 0.009
14	Dehydroevodiamine	0.902 ± 0.041	0.684 ± 0.031
15	Quercetin	/	0.069 ± 0.003
16	Glycyrrhizic acid	/	0.121 ± 0.005
17	Limonin	0.295 ± 0.013	0.353 ± 0.016
18	Evodiamine	0.426 ± 0.019	0.579 ± 0.028
19	Rutaecarpine	0.278 ± 0.012	0.510 ± 0.025
20	Glycyrrhetinic acid	/	0.044 ± 0.002
21	Evocarpine	0.401 ± 0.020	0.462 ± 0.022
22	1-methyl-2- (6Z,9Z)-10-penta-decadinenyl-4(1H)-quinolone	/	/
23	Dihydroevocarpine	0.069 ± 0.003	0.116 ± 0.007

/: Not quantitative or detected.

The oil yield was 0.60 ml per 100 g CEF, and 0.40 ml per 100 g LPEF. A total of 48 compounds in volatile oil were identified based on the MS database NIST11 (Gaithersburg, United States), and their relative contents were determined according to the normalization method (Li et al., 2017). The total amount of volatile oil decreased significantly, but its composition and proportion had no significant variation after processing.

### General Behavior of Animals

During the 15 days of administration, the rats in the control group had normal activities and bright hair color, drinking and eating were normal, and no death was recorded. The model group gradually returned to normal, and there was no significant difference compared with the control group after 15 days. All the low-dose groups and three dose groups of VO had normal diet, feces, and activities. The rats in high-dose groups of CEF and LPEF had black and hard stool; in addition, they had poor appetite, drank less water, and were unwilling to move. Some rats in the medium-dose group also showed the above condition. More detailed information is summarized in Supplementary Table S2.

### Body Weight and Liver/Body Weight Ratio

**FIGURE 3:**
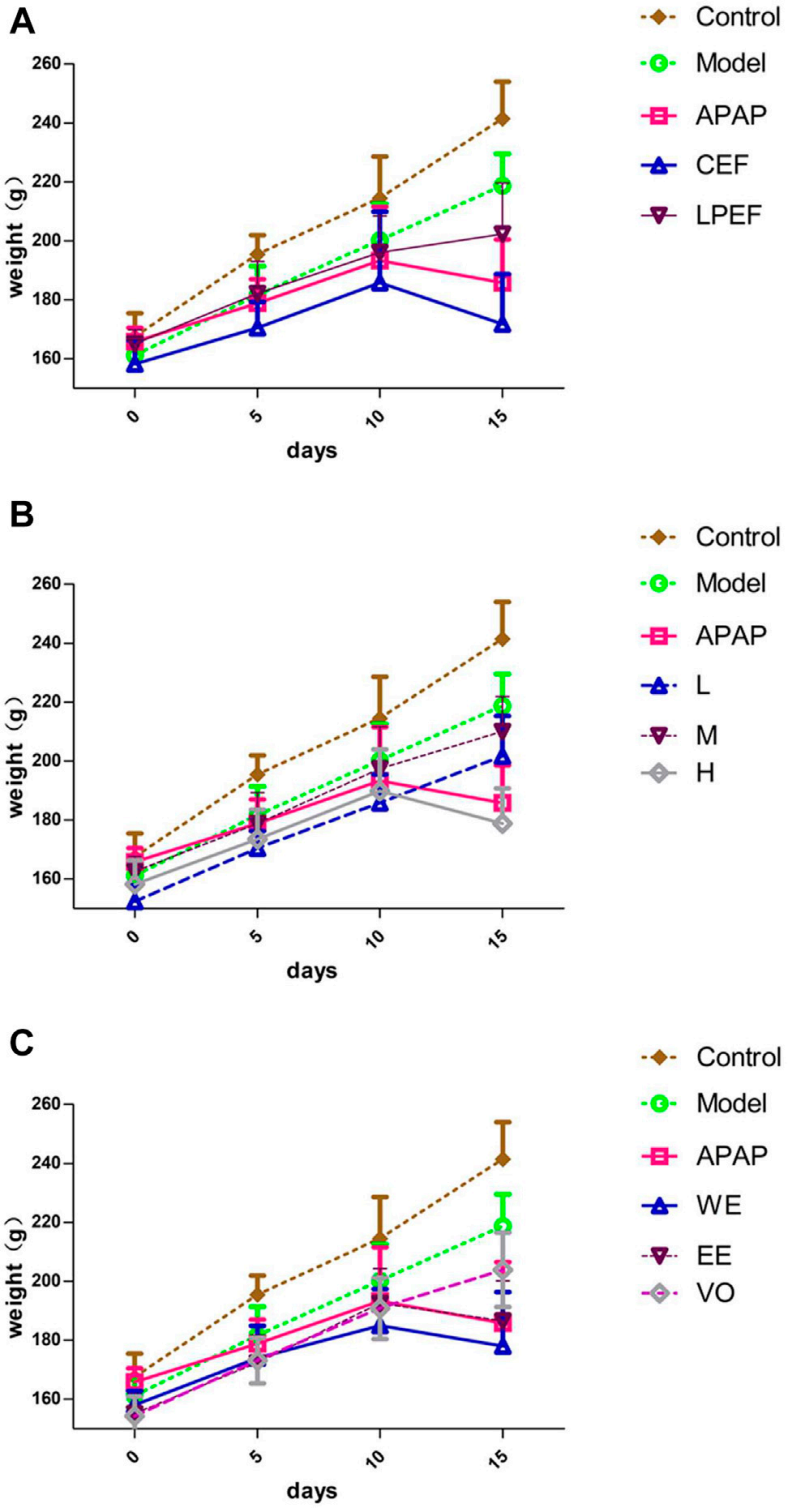
Changing trends of body weight of various groups. **(A)** WE at high dose in CEF and LPEF. **(B)** Three doses of WE of CEF. **(C)** Different extracts at high dose in LPEF. Values are mean ± SD of 10 replicated samples.

The changes in body weight can be intuitively seen in Figure 3. Control group, model group, and low-dose groups kept gaining weight over the 15 days, and the weight-gain order from heavy to light was as follows: control, model, low-, medium-, and high-dose groups (Figure 3). The high-dose groups underwent a process of weight gain and then weight loss (Figure 3A). On the whole, the weight gain of the LPEF group was greater than that of the CEF group when given the same doses (Figure 3A). No significant differences were observed within the first 10 days among the different doses (L, M, and H; Figure 3B) and extracts (WE, EE, and VO; Figure 3C). On day 15, the body weight of each group from heavy to light was as follows: low, medium, and high (VO, EE, and WE). More detailed data and statistical results can be accessed in Table 4.

**TABLE 4:** Comparison of body weight (BW), liver weight (LW), and liver-to-body weight ratio among different groups.

Group		Body weight (g)						Liver weight (g) (15th day)	Ratio of LW/BW (%)
Drug	Extract	^a^Dose	Initial	Increment (0–5 days)	Increment (0–10 days)	Increment (0–15 days)	15th day		
Control	/	/	193.2 ± 11.5	27.9 ± 3.4	47.5 ± 4.2	73.9 ± 8.2	262.4 ± 14.1	6.901 ± 0.516	2. 663 ± 0.085
Model	/	/	181.1 ± 12.9	20.5 ± 1.8	39.1 ± 3.8	67.6 ± 5.8	251.3 ± 10.7	6.601 ± 0.731	2.656 ± 0.102
APAP	/	0.21	175.8 ± 13.7	13.4 ± 1.5	27.5 ± 2.6	20.4 ± 2.1**	201.8 ± 11.9	8.873 ± 0.785	4.441 ± 0.203**
		L	187.3 ± 10.5	18.1 ± 2.3** ^**◇◇**^	35.5 ± 3.3** ^**◇◇**^	50.4 ± 5.4** ^**◇◇**^	239.3 ± 12.1	6.874 ± 0.583	2.867 ± 0.102^**◇◇**^
	WE	M	172.5 ± 13.1	16.2 ± 1.9** ^**◇◇**^	34.8 ± 3.1** ^**◇◇**^	47.5 ± 4.9** ^**◇◇**^	221.8 ± 15.4	8.464 ± 0.774	3.841 ± 0.155* ^**◇**^
		H	181.2 ± 8.1	15.3 ± 1.7** ^**◇◇**^	31.6 ± 2.8** ^**◇◇**^	20.6 ± 1.7**	202.8 ± 9.3	9.002 ± 0.811	4.265 ± 0.233**
		L	180.0 ± 15.4	20.9 ± 2.3** ^**◇◇**^	36.4 ± 3.7** ^**◇◇**^	42.1 ± 3.9** ^**◇◇**^	223.7 ± 18.4	6.361 ± 0.439	2.845 ± 0.206^**◇◇**^
CEF	EE	M	184.5 ± 13.6	19.5 ± 2.2** ^**◇◇**^	35.8 ± 3.6** ^**◇◇**^	49.6 ± 5.1** ^**◇◇**^	235.6 ± 11.8	8.774 ± 0.681	3.641 ± 0.108** ^**◇**^
		H	179.8 ± 12.9	16.3 ± 1.4** ^**◇◇**^	32.2 ± 3.4** ^**◇◇**^	26.2 ± 2.2** ^**◇◇**^	207.7 ± 13.1	8.787 ± 0.786	4.102 ± 0.266**
		L	180.6 ± 14.5	20.4 ± 2.1** ^**◇◇**^	38.2 ± 4.1* ^**◇◇**^	54.5 ± 5.4** ^**◇◇**^	236.5 ± 16.2	6.713 ± 0.642	2.868 ± 0.169^**◇◇**^
	VO	M	182.4 ± 12.7	19.8 ± 1.7** ^**◇◇**^	38.8 ± 3.7* ^**◇◇**^	60.1 ± 6.2* ^**◇◇**^	241.4 ± 17.5	7.724 ± 0.597	3.198 ± 0.121^**◇◇**^
		H	184.8 ± 13.4	17.1 ± 1.8** ^**◇◇**^	31.2 ± 3.0** ^**◇◇**^	39.2 ± 4.1** ^**◇◇**^	225.2 ± 14.7	9.344 ± 0.758	3.983 ± 0.105**
		L	182.9 ± 14.9	19.7 ± 2.2** ^**◇◇**^	27.2 ± 2.5** ^**◇◇**^	44.6 ± 4.4** ^**◇◇**^	228.7 ± 16.2	6.623 ± 0.715	2.796 ± 0.147^**◇◇**^
	WE	M	181.6 ± 13.2	17.3 ± 1.6** ^**◇◇**^	35.8 ± 3.6** ^**◇◇**^	50.2 ± 5.2** ^**◇◇**^	233.8 ± 17.3	7.385 ± 0.672	3.147 ± 0.185* ^#**◇**^
		H	185.1 ± 14.9	16.5 ± 1.4** ^**◇◇**^	33.7 ± 3.1** ^**◇◇**^	37.2 ± 3.9** ^**##◇◇**^	224.1 ± 16.5	9.066 ± 0.729	3.894 ± 0.279** ^##^
		L	183.3 ± 14.8	21.1 ± 2.0** ^**◇◇**^	39.1 ± 3.8* ^**◇◇**^	55.1 ± 5.9** ^**◇◇**^	239.6 ± 17.2	7.293 ± 0.653	2.917 ± 0.193^**◇◇**^
LPEF	EE	M	178.1 ± 10.4	18.8 ± 1.7** ^**◇◇**^	35.4 ± 3.5** ^**◇◇**^	48.8 ± 4.3** ^**◇◇**^	226.5 ± 14.6	7.394 ± 0.713	3.209 ± 0.174* ^#**◇**^
		H	185.1 ± 8.7	17.2 ± 1.5** ^**◇◇**^	34.4 ± 3.2** ^**◇◇**^	43.4 ± 4.0** ^**##◇◇**^	230.4 ± 16.3	8.436 ± 0.751	3.598 ± 0.237** ^#^
		L	186.5 ± 12.2	23.1 ± 2.7** ^**◇◇**^	44.4 ± 4.2^**◇◇**^	61.8 ± 5.9* ^**◇◇**^	248.2 ± 15.3	6.804 ± 0.619	2.665 ± 0.116^**◇◇**^
	VO	M	183.3 ± 9.9	22.6 ± 2.2** ^**◇◇**^	45.3 ± 4.5^**#◇◇**^	51.7 ± 4.8** ^**◇◇**^	235.6 ± 10.9	7.227 ± 0.613	2.966 ± 0.108^**◇◇**^
		H	184.3 ± 8.5	18.8 ± 1.9** ^**◇◇**^	36.5 ± 3.9** ^**#◇◇**^	49.6 ± 4.7** ^**##◇◇**^	234.6 ± 15.7	8.157 ± 0.733	3.345 ± 0.184** ^##**◇**^

a Doses are similar to that in Table 1. APAP, 0.21 mg kg^−1^; L, M, and H: 1.05,5.25, and 10.5 g kg^−1^, respectively.

Values are mean ± SD of 10 replicated samples; vs. control group, *p* < 0.05 (*) and *p* < 0.01 (**); vs. CEF, *p* < 0.05 (^**#**^) and *p* < 0.01 (^**##**^); vs. APAP, *p* < 0.05 (^**◇**^) and *p* < 0.01 (^**◇◇**^).

The ratio of liver/body weight (also called liver coefficient) increased with doses going up (Table 4). Compared to the control group, most of the dose groups had greater liver coefficients, while no significant difference was observed between the low-dose group and the control group (^*^ *p* < 0.05). Liver coefficients of the LPEF groups were less than those of the CEF groups with significant difference (^#^ *p* < 0.05 or ^##^ *p* < 0.01).

### Effects on Mitochondrial ATP Enzymes in Rat Liver

**FIGURE 4:**
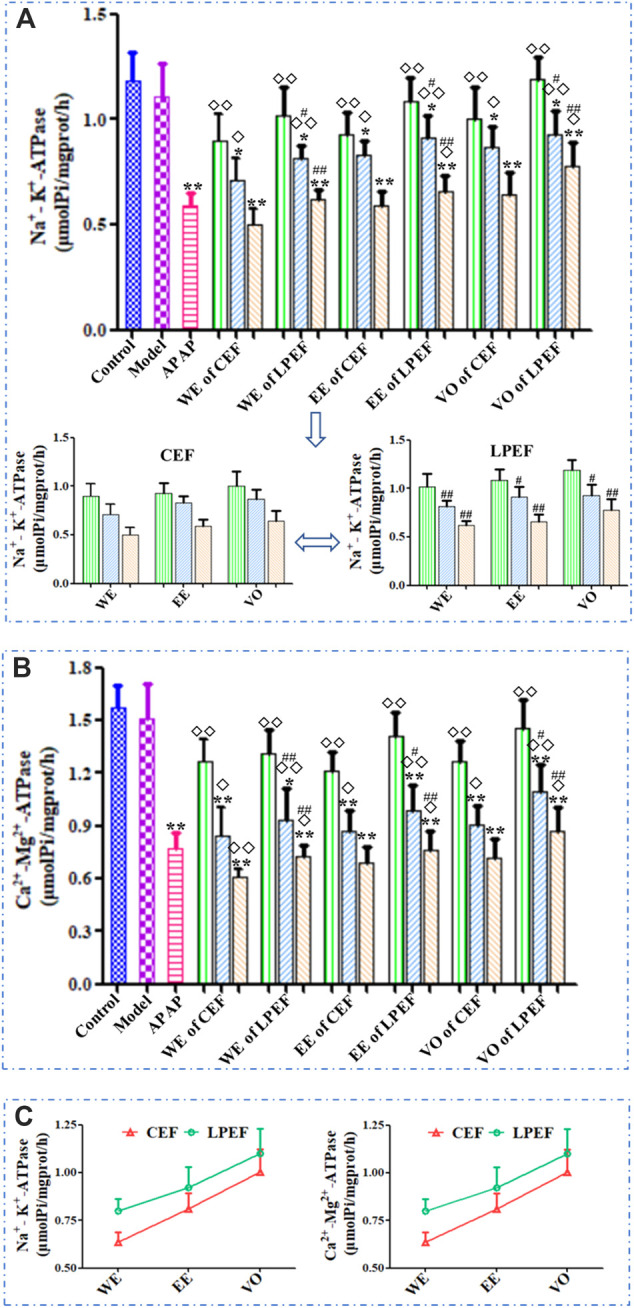
Comparison of mitochondrial ATP enzyme activities in the liver of rats treated with different extracts. **(A)** Na^+^-K^+^-ATPase (upper: comparison between 21 groups; lower: magnified comparison between CEF and LPEF). **(B)** Ca^2+^-Mg^2+^-ATPase and **(C)** changing trend of ATP enzyme activities between different extracts at high dose. Values are mean ± SD of 10 replicated samples; vs. control group, *p* < 0.05 (*) and *p* < 0.01 (**); vs. CEF, *p* < 0.05 (^**#**^) and *p* < 0.01 (^**##**^); vs. APAP, *p* < 0.05 (^**◇**^) and *p* < 0.01 (^**◇◇**^). 10.5 g kg^−1^, 5.25 g kg^−1^, 1.05 g kg^−1^.

As shown in Figure 4 and Supplementary Table S3, the effects of various drugs on each mitochondrial ATP enzyme were similar, and all the drugs (APAP, CEF, and LPEF) attenuated the enzyme activity in a dose-dependent manner. Compared to the control group, high and medium doses of CEF and LPEF significantly attenuated the activity of Na^+^-K^+^-ATPase and Ca2^+^-Mg2^+^-ATPase (*p < 0.05 or **p < 0.01) in the liver, while the effects of the low-dose group were not significant. Significant or very significant differences (^◇^ *p* < 0.05 or ^◇◇^ *p* < 0.01) can be observed between EF (CEF and LPEF) and the positive control groups (APAP). In comparison with the corresponding CEF groups (lower graph of Figure 4A), three doses of LPEF groups significantly improved the activity of ATP enzymes (^#^ *p* < 0.05 or ^##^ *p* < 0.01). Figure 4C showed the effects of various extracts on the ATP enzymes. The influence in a descending order was as follows: WE, EE, and VO. In addition, the difference between the effects of CEF and LPED on ATP enzymes can be seen more intuitively in Figure 4C.

### Effects on Serum Enzymes in Rats

**FIGURE 5:**
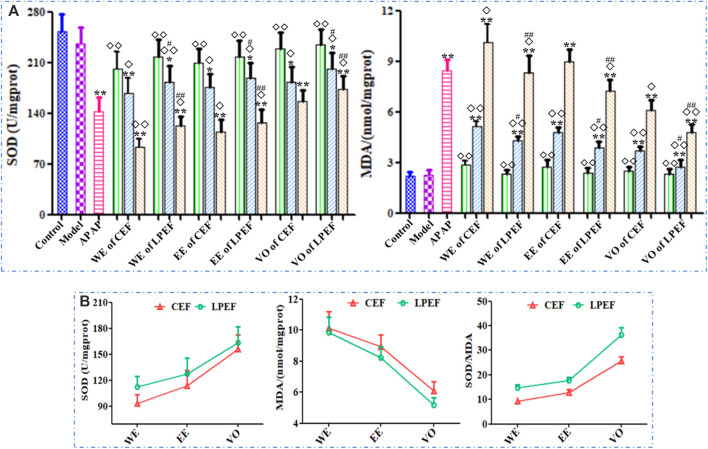
Effects of 21 groups of samples on serum SOD and MDA activities **(A)**, and comparison of SOD, MDA activities, and SOD/MDA between different extracts at high dose **(B)**. Values are expressed as mean ± SD, *n* = 10; vs. control group, *p* < 0.05 (*) and *p* < 0.01 (**); vs. CEF, *p* < 0.05 (^**#**^) and *p* < 0.01 (^**##**^); vs. APAP, *p* < 0.05 (^**◇**^) and *p* < 0.01 (^**◇◇**^). 10.5 g kg^−1^, 5.25 g kg^−1^, 1.05 g kg^−1^.

As shown in Figure 5 and Supplementary Table S4, the SOD level in rat serum decreased while the dose was increasing; the MDA content increased in a dose-dependent manner. The MDA content in the high- and medium-dose groups significantly increased and SOD content reduced compared with those in the control group (*p < 0.05 or **p < 0.01); the effect of the low-dose group was not significant. Compared with CEF, LPEF significantly increased the SOD level and reduced the MDA content in serum (^#^ *p* < 0.05 or ^##^ *p* < 0.01). The effects of different extracts on MDA and SOD were in the following order: WE > EE > VO (Figure 5B). The differences between CEF and LPEF, also among three extracts, can be amplified with the SOD/MDA plot (Figure 5B), which indicated that the SOD/MDA was a more sensitive index for liver damage.

**FIGURE 6:**
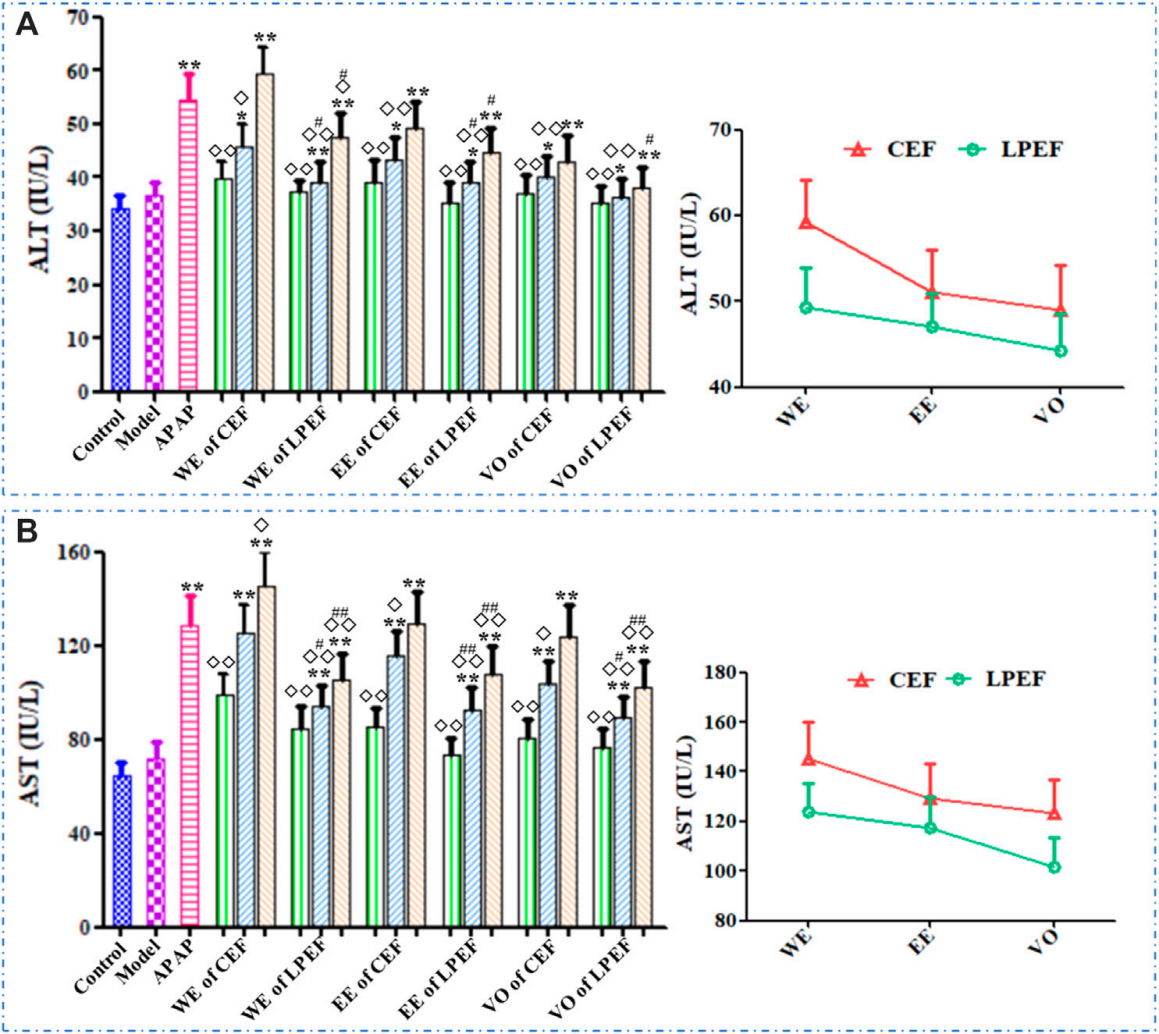
Effects on serum ALT **(A)** and AST **(B)** activities of different drugs. Left graph: comparison between 21 groups; right graph: comparison between three CEF and LPED extracts at high dose. Values are mean ± SD of 10 replicated samples; vs. control group, *p* < 0.05 (*) and *p* < 0.01 (**); vs. CEF, *p* < 0.05 (^**#**^) and *p* < 0.01 (^**##**^); vs. APAP, *p* < 0.05 (^**◇**^) and *p* < 0.01 (^**◇◇**^). 10.5 g kg^−1^, 5.25 g kg^−1^, 1.05 g kg^−1^.

Figure 6 illustrated the serum ALT and AST results of various groups. The ALT and AST levels in rat serum increased with the doses going up. The AST and ALT levels in the high- and medium-dose groups significantly increased (*p < 0.05 or **p < 0.01) compared with those in the control group. Significant or very significant differences can be observed between the groups of EF and APAP (^**◇**^ *p* < 0.05 or ^**◇◇**^ *p* < 0.01). In comparison with the similar dose of CEF, LPEF reduced the ALT and AST levels in serum significantly (^#^ *p* < 0.05 or ^##^ *p* < 0.01). As can be seen from Figure 6, the effects of different extracts on ALT and AST were in the following order: WE > EE > VO. More specific data can be seen in Supplementary Table S5.

**FIGURE 7:**
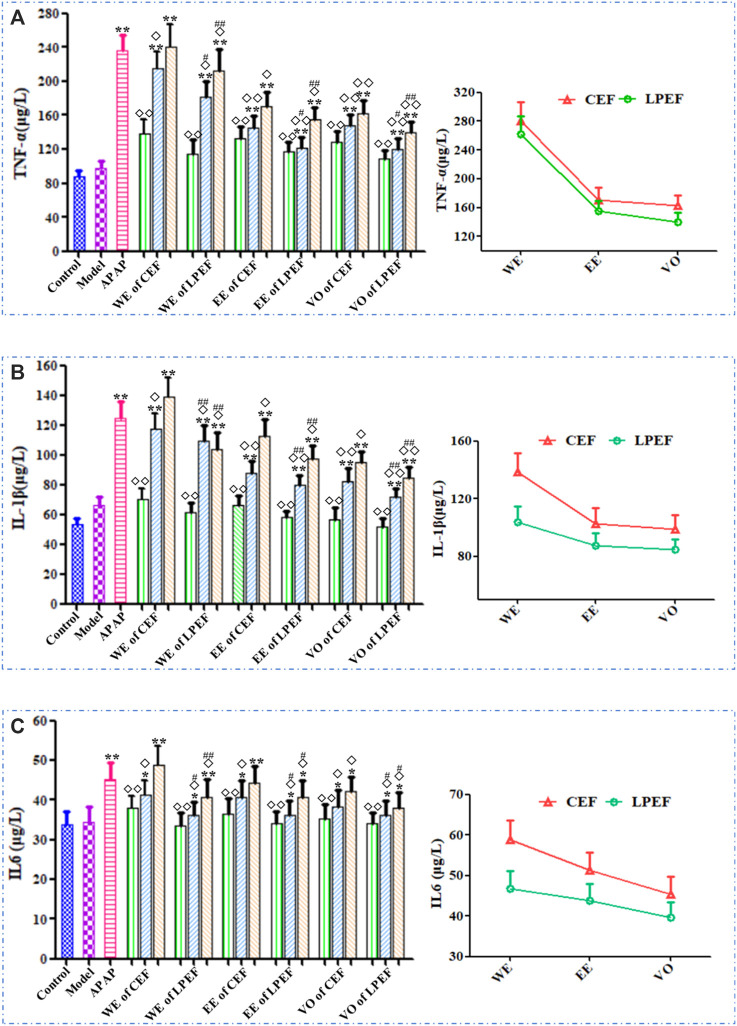
Effects on serum inflammatory factors of different drugs: TNF-*α* **(A)**, IL-1*β* **(B)**, and IL-6 **(C)**. Left graph: comparison between 21 groups; right graph: comparison between three CEF and LPED extracts at high dose. Values are mean ± SD of 10 replicated samples; vs. control group, *p* < 0.05 (*) and *p* < 0.01 (**); vs. CEF, *p* < 0.05 (^**#**^) and *p* < 0.01 (^**##**^); vs. APAP, *p* < 0.05 (^**◇**^) and *p* < 0.01 (^**◇◇**^). 10.5 g kg^−1^, 5.25 g kg^−1^, 1.05 g kg^−1^.

The effects of various drugs on the serum TNF-*α*, IL-1*β*, and IL-6 are shown in Figure 7 and Supplementary Table S6. In general, the TNF-*α*, IL-1*β*, and IL-6 levels in rat serum increased with the doses going up, and their trend was basically the same. The high and medium doses of CEF and LPEF significantly increased the levels of TNF-*α*, IL-1*β*, and IL-6 (*p < 0.05 or**p < 0.01) in comparison with the control group, while the low-dose group had no statistical significance. Compared with CEF, the corresponding dose of LPEF significantly reduced the levels of TNF-*α*, IL-1*β*, and IL-6 in serum (^#^ *p* < 0.05 or ^##^ *p* < 0.01). Various extracts had effects on the above inflammatory factors, and the order of influence was WE > EE > VO. The differences among three extracts, also CEF and LPEF, can be seen more intuitively from the right graph of Figure 7.

### Bax, Bcl-2, and Caspase 3 Protein Expressions in Rats

**FIGURE 8:**
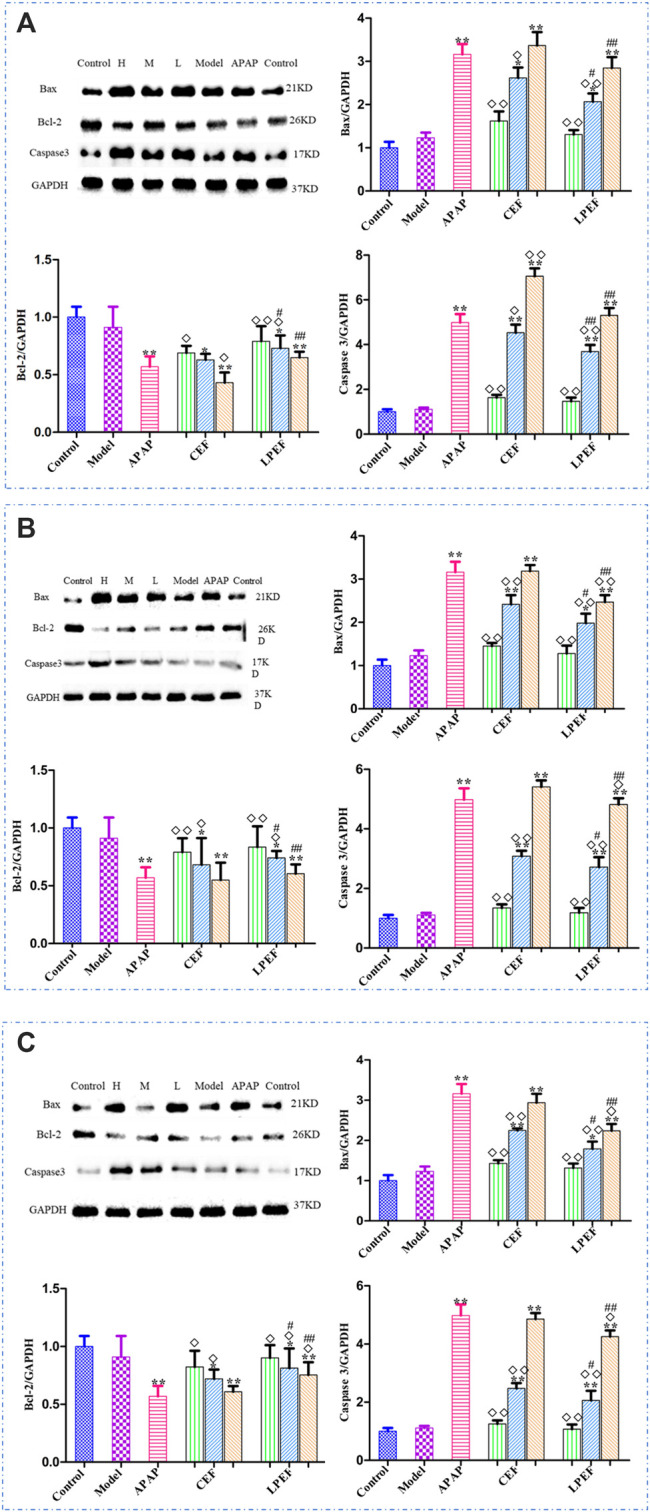
Effects of different drugs on apoptosis-associated proteins in rats with stomach excess-cold syndrome: WE **(A)**, EE **(B)**, and VO **(C)**. Values are mean ± SD of three replicated sample; vs. control group, *p* < 0.05 (*) and *p* < 0.01 (**); vs. CEF, *p* < 0.05 (^**#**^) and *p* < 0.01 (^**##**^); vs. APAP, *p* < 0.05 (^**◇**^) and *p* < 0.01 (^**◇◇**^). 10.5 g kg^−1^, 5.25 g kg^−1^, 1.05 g kg^−1^.

The protein expressions in livers of rats treated with various drugs are shown in Figure 8. On the whole, the expressions of Bax and Caspase 3 increased rapidly with the doses going up, and the Bcl-2 expression decreased. There was no significant difference in protein expression between the low-dose group and the control group. The expressions of Bax and Caspase 3 in the LPEF groups significantly decreased, and the Bcl-2 expression increased in comparison with that in the CEF groups (^#^ *p* < 0.05 or ^##^ *p* < 0.01). As shown in Figure 8D, the effects of various extracts on the protein expressions in rat livers were in the following order: WE > EE > VO.

### Effect on Liver Pathology of Rats

**FIGURE 9:**
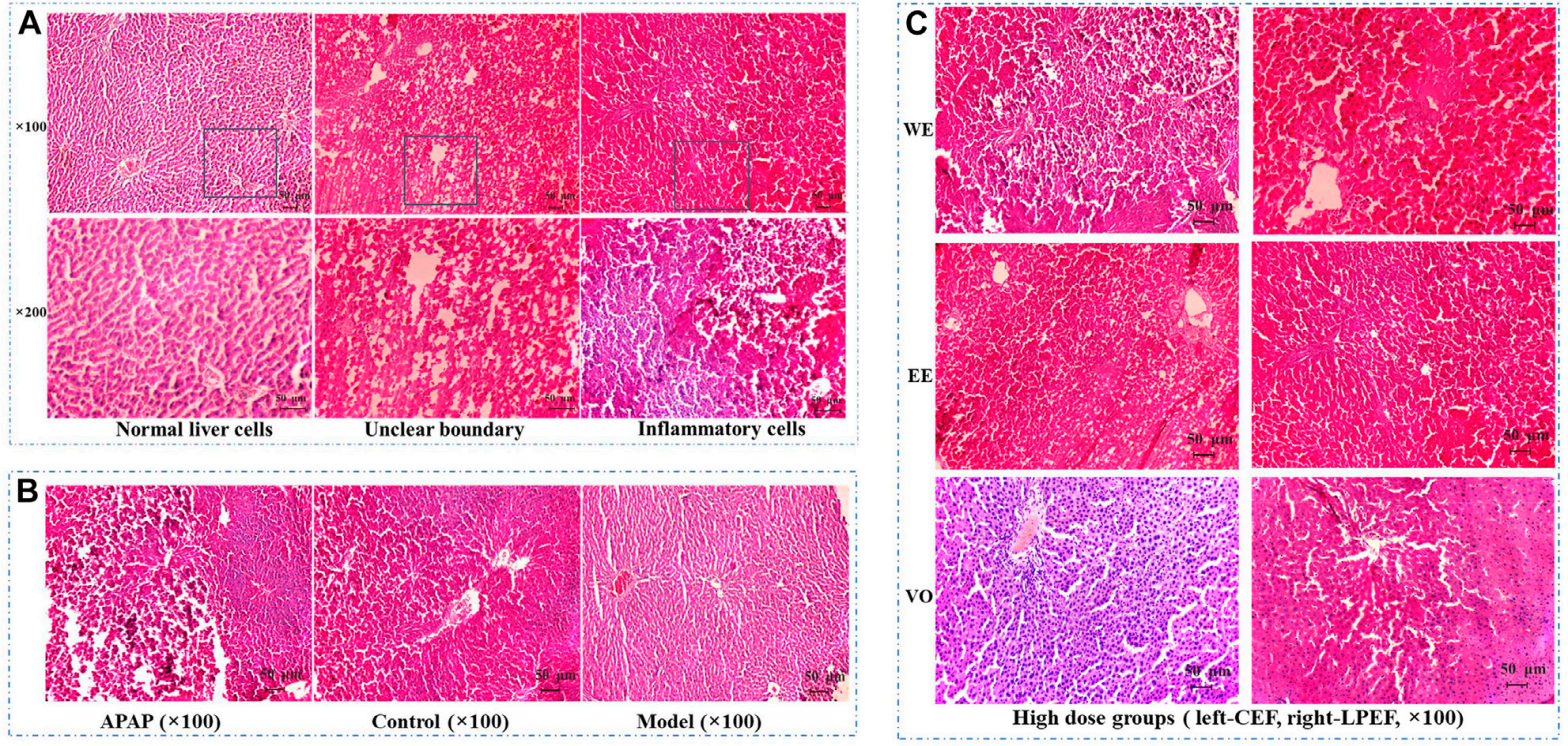
Effects of CEF and LPEF on pathomorphology of liver issue in rats with stomach excess-cold syndrome treated with different extracts. **(A)** Normal liver cells and hepatic damage cells. **(B)** Comparison of APAP group, model group, and control group. **(C)** High dose groups.

After HE staining, the results were observed under an electron microscope (Figure 9). As can be seen from the figures, the liver cells of the model group, control group, and low-dose groups (Supplementary Figure S1) showed uniform size, cord-like distribution, and clear visible nuclei (Figure 9B). However, in the medium-dose groups (Supplementary Figure S1), cells were shrinking, the edge structure was unclear, and the arrangement of hepatocytes was slightly disordered. In the high-dose groups (Figure 9C), cells were shrinking, cell volume became smaller, and the hepatocyte cords were arranged disorderly. For the APAP group (Figure 9B), the boundaries between cells were not clear, cells were soaked by inflammatory cells, and some were even necrotic. In the WE and EE high-dose groups in CEF, there were explicit lesions and necrosis, accompanied by inflammatory cell immersion, while the change of liver cells in the VO high-dose group was smaller. In addition, the above hepatotoxic characteristics of LPEF groups were obviously milder than those of CEF (Figure 9C and Supplementary Figure S1). Through the pathological examination of the liver tissue in the target area of CEF and LPEF, it suggested that liver damage was becoming more and more serious with the increase of dosage. Liver damage caused by water extract was the most serious among three different extracts, and damage could be greatly alleviated by processing.

### Summary of Hepatotoxicity Experiments

Some hepatotoxicity results can be summarized as follows: ① The toxicity of EF (including CEF and LPEF) showed a dose-dependent relationship, and no significant differences were found between the control group and low-dose groups. ② Compared with the corresponding CEF groups, the medium dose and high dose of LPEF groups showed lower toxicity (^#^ *p* < 0.05 or ^##^ *p* < 0.01). ③ All the three extracts had definite hepatotoxicity, and the toxicity was in the descending order of WE, EE, and VO. Taking the high dose of WE groups for example, the changing trends and significance among various groups are shown in Table 5, and similar conclusions can be obtained with the above summary.

**TABLE 5:** Changing trends and significance results of the effects of the drugs on the toxicological indexes, taking the high-dose groups of CEF and LPEF water extracts (WE) for example.

Evaluating index	Comparison between the high dose of WE and the other groups			
	CEF vs*.* control	LPEF vs*.* CEF	WE vs*.* VO in CEF	High dose vs*.* low dose in CEF
ATPases	↓↓	↑↑	↓↓	↓↓
AST	↑↑	↓↓	↑↑	↑↑
ALT	↑	↓↓	↑↑	↑↑
SOD	↓↓	↑↑	↓↓	↓
MDA	↑↑	↓↓	↑↑	↑↑
TNF-*α*	↑↑	↓↓	↑↑	↑↑
IL-6	↑	↓↓	↑↑	↑
IL-1*β*	↑↑	↓↓	↑↑	↑↑
Bax	↑↑	↓↓	↑↑	↑↑
Bcl-2	↓↓	↑↑	↓↓	↓↓
Caspase 3	↑↑	↓↓	↑↑	↑↑

↑, significant increase; ↑↑, very significant increase.

↓, significant decrease; ↓↓, very significant decrease.

## Discussion

In recent years, there have been increasing reports of hepatotoxicity of traditional medicine, which has seriously affected the reputation of TCM and has become a key bottleneck that restricts its clinical efficacy and development. EF, as a commonly used medicinal herb, has a good therapeutic effect, but its toxicity restricts its wider clinical practice to some extent. The hepatotoxicity of EF is one of the most concerned clinical adverse reactions. Therefore, the study of toxic fraction, components, dose, and mechanism, and the scientific connotation of detoxification by drug processing can help to better guide the clinical use of EF.

The toxicity of TCM is closely related to its efficacy and syndromes; the comprehensive evaluation and cognition of toxicity should be based on the syndromes of TCM. There are some certain differences in efficacy and toxicity between different diseases and syndrome models. Therefore, the toxicity of a drug should be reasonably evaluated and scientifically recognized under the background of syndrome. To date, only two reports (Li and Sun, 2015a; Li and Sun, 2015b) regarding the toxicity of CEF are available; however, the relevant model is the stomach deficiency-cold syndrome. According to the TCM theory, EF was used to treat stomach excess-cold syndrome. The main pathogenesis of this syndrome is the invasion of the stomach by cold evil. The course of stomach excess-cold syndrome is short, and the onset is usually sudden. The pathogenic factors of cold are external, and the main pathogenesis is cold (han in TCM, noun) coagulation and gas (qi in TCM) obstruction. The epigastrium is usually severe pain and refusal to be pressed. The pharmacological effects (analgesic, anti-inflammatory, and anti-ulcer) of EF are exactly counteracting the above symptoms. This study, therefore, established a stomach excess-cold syndrome model in rats by intragastric administration of a large dose of cold *Zhimu* to damage gastric mucosa according to the literature (Qin et al., 2013). The model was designed in accordance with the traditional etiological theory and in line with the “warm medicine” characteristics of EF in the TCM theory. In the experiments, the model “stomach excess-cold syndrome” was found to be acute, its pathological process was short, and the symptoms could be self-healing after the discontinuation of Zhimu. Furthermore, EF could increase intracellular cAMP level and adjust cAMP/cGMP level in rats with stomach excess-cold syndrome; thus, rats could restore their normal stomach function more quickly. According to general behavior, gastrointestinal functions (Table 2), the pathological process (Figure 1), and the changes of biochemical indicators, the animal model established in this work well simulated the stomach excess-cold syndrome and is suitable for the hepatotoxic research of EF and LPEF.

The multiple functions of the liver make it best to use multiple approaches for evaluating the hepatic injury and changes to function. Serum ALT and AST are the most commonly used pathology indexes in the hepatotoxicity experiments (Zhou et al., 2011; Sun et al., 2017; Ren et al., 2020), and sometimes ALP and LDH are detected (Liu et al., 2018; Zhang et al., 2018). Some slightly more in-depth studies investigated the changes in MDA, SOD, NO, and GSH-Px (Huang et al., 2012; Li and Sun, 2015a; Li and Sun, 2015b; Liu et al., 2018). As the research goes on, the protein expression and inflammatory cytokines have also been used to assess hepatic injury (Liao et al., 2015; Liu et al., 2018). In order to evaluate the hepatotoxicity of CEF and LPEF more systematically and comprehensively, we introduced more detection methods and toxicological indexes, such as monitoring general behavior and the ratio of liver to body weight, the biochemical detection of serum enzymes (SOD, MDA, ALT, and AST), mitochondrial ATP enzymes and inflammatory factors (TNF-*α*, IL-1*β*, and IL-6), protein expression (Bax, Bcl-2, and Caspase 3 protein), and histomorphology detection. The mitochondrial ATP enzymes in rat liver were found to be the most sensitive indicator to indicate liver injury; very minor injuries can be monitored with the ATP enzymes in our experiments. With the progress of liver damage, the changes of serum enzymes and inflammatory factors began to become significant. Finally, the liver damage can be intuitively observed by histomorphology analysis. Based on the above toxicological evaluation approaches and indicators, a possible and simplified toxic mechanism can be deduced: the toxic mechanism of EF is related to peroxidation damage, inflammatory reaction factor, and mitochondrial injury, and some toxic compounds in EF produce drug–protein adducts resulting in immune liver damage.

The screening of toxic components in natural products generally follows a process from the extract to a single component, and the identification of the toxic fraction is the first key step. Previous studies have shown that WE, EE, and VO of EF can cause liver damage in animals, which is manifested with elevated serum ALT and AST levels, fatty degeneration, and necrosis of liver tissue (Huang et al., 2012; Huang and Sun., 2012; Sun et al., 2012; Zhu et al., 2013; Li and Sun, 2015a; Li and Sun, 2015b; Liang et al., 2017; Zhao et al., 2020). However, only one report compared the toxicity of various EF extracts and concluded that VO had the strongest acute toxicity followed by the total extract EE and WE (Huang et al., 2010). Up to now, no report has been retrieved about the comparison of the hepatotoxicity of the different fractions of EF. In this work, All the three extracts of EF were found to be toxic, and the hepatotoxicity was in the descending order of WE, EE, and VO, which is inconsistent with the literature about the acute toxicity (Huang et al., 2010; Sun et al., 2015). There are two possible reasons for the differences: different animal species and syndrome model. The rats with stomach excess-cold syndrome were used in this work, but normal mice were tested in Huang’s experiments. Very recently, more studies paid attention to the hepatotoxicity of WE (Huang et al., 2012; Huang and Sun., 2012; Zhou et al., 2013), which also confirmed our results from the side. Similar to that, the toxic fraction of EF was still under debate, and the main hepatotoxic components were also not clear. Li et al. claimed that 50% ethanol extract (concluding alkaloids, triterpenoids, and flavonoids) was responsible for the hepatotoxicity of EF (Li et al., 2017). Some studies speculated that isomers of caffeoyl gluconic acid could be the hepatotoxic substance (Wang et al., 2017; Wang et al., 2019). Our previous study found that caffeoyl gluconic acids, coniferin, and some quinolone alkaloids probably should be associated with the hepatotoxicity of EF (Liang et al., 2017; Chen et al., 2019). After processing with licorice, the main chemical change of EF was the significant reduction in some organic acids (Figure 2 and Table 2). The organic acids mainly include chlorogenic acids, caffeoyl gluconic acids, and their isomers, which mainly are present in aqueous extracts. The water extract of EF was found to be the most toxic in this work. Chlorogenic acids have a partially similar structure to caffeoyl gluconic acids, and their content significantly decreased after processing. However, chlorogenic acids may not be a hepatotoxic compound according to the current results (Liang et al., 2017; Li, 2020). Therefore, we could draw a relatively reliable conclusion that caffeoyl gluconic acids and their isomers might be the main hepatotoxic components in the water extract of EF based on the above discussion and the current experimental results. However, the ethanol extract and volatile oil of EF were also found to have definite hepatotoxicity, which suggested that there must be other hepatotoxic compounds in EF. In combination with our previous conclusions (Li et al., 2017; Liang et al., 2017), we deduce that the quinolone alkaloids and ocimene isomers are very likely to be the main hepatotoxic components in methanol extract and volatile oil of EF, respectively. In summary, it was indicated that the hepatotoxicity of EF should be the joint action of many toxic components, and specific toxic components remain to be studied systematically.

It has been reported that the acute toxicity of EF is dose-dependent (Huang et al., 2012; Sun et al., 2012). In this work, the hepatotoxicity of EF was found to be dose-dependent on various approaches and toxicology indexes. For an adult, the range of clinical dose of EF is very wide with 1–70 g/day, and the pharmacopeial dose is 2–5 g/day. The low dose in this work was set at 1.05 g/kg/day, and equivalent to 10 g/day for an adult. Fortunately, no significant hepatotoxicity was observed for the low dose of CEF and LPEF, which indicates that the pharmacopoeia dose may be a safe one during short-term ingestion of CEF or LPEF. It can be inferred that most cases of toxic and side effects of EF may be generally caused by long-term use of large doses.

Chinese medicine has noted the toxicity of EF since ancient time, and EF is marked with “mild toxic” and “toxic” in TCM books. Drug processing and compatibility are often used to reduce or eliminate its toxic and side effects. Licorice processing is one of the commonly used processing techniques for CEF. After processing with licorice, a series of physical, chemical, and biological changes occurred in EF, in which the contents of organic acids significantly decreased and quinolone alkaloids increased a little. As interpreted above, caffeoyl gluconic acids and quinolone alkaloids are potential hepatotoxic components in EF. The increase and decrease of toxic components are closely related to the detoxification mechanism of drug processing. In addition, licorice is good at moderating all kinds of toxic herbs probably through antagonism. Liquiritin and glycyrrhizic acid in licorice have been reported to have detoxification effect (Wu et al., 2020; Xiao et al., 2021). Accordingly, the hepatotoxicity of LPEF is significantly lower than that of CEF. We speculate that licorice processing combines the effects of drug processing and compatibility, and further studies on the material basis and mechanism of detoxification are underway.

## Conclusion

In this work, the hepatotoxicity of different extracts of CEF and LPEF at different doses was compared comprehensively based on the stomach excess-cold syndrome model with multiple toxicological evaluation approaches and indicators. All three extracts of EF (including CEF and LPEF) have dose-dependent hepatotoxicity, and the toxicity was in the descending order of WE, EE, and VO. The toxic mechanism of EF may be related to peroxidation damage, inflammatory reaction factor, and mitochondrial injury. The hepatotoxicity of CEF can be significantly reduced by licorice toasting. EF should be safe for short-term use at pharmacopeial dose under the guidance of the TCM theory. The detoxification mechanism may be related to the reduction of toxic components and antagonistic action of licorice, and the more specific material basis and mechanism of detoxification need to be further studied.

## Data Availability

The original contributions presented in the study are included in the article/Supplementary Material. Further inquiries can be directed to the corresponding author.
