# Supplementary material for: Hepatotoxicity Comparison of Crude and Licorice-Processed Euodiae Fructus in Rats With Stomach Excess-Cold Syndrome
Source: Front Pharmacol. 2021 Nov 23;12:756276. doi: 10.3389/fphar.2021.756276 (PMC8650065; doi:10.3389/fphar.2021.756276)
Supplement: Supplementary file 1 [file Table1.docx]

**Table S1 |** Q-TOF/MS data of the 23 detected compounds.

| **Peak No.** | **Compound** | **Formula** | **Ion mode** | **MW**  **(Da)** | **Measured**  **(Da)** | **Error (ppm)** | **Product ion (m/z)** |
| --- | --- | --- | --- | --- | --- | --- | --- |
| 1 | trans-caffeoylgluconic acid-d1 | C_15_H_18_O_10_ | [M-H]^-^ | 358.0899 | 357.0857 | 3.4 | 195.0527;129.0218;75.0141;179.0365;99.0120 |
| 2 | trans-caffeoylgluconic acid-d2 | C_15_H_18_O_10_ | [M-H]^-^ | 358.0899 | 357.0855 | 3.4 | 195.0532;179.0366;129.0218;99.0119;75.0141 |
| 3 | trans-caffeoylgluconic acid-d3 | C_15_H_18_O_10_ | [M-H]^-^ | 358.0899 | 357.0852 | 3.4 | 195.0528;179.0361;135.0471;129.0217;99.0119;75.0134 |
| 4 | Neochlorogenic acid | C_16_H_18_O_9_ | [M-H]^-^ | 354.0951 | 353.0908 | 3.3 | 191.0577;179.0365;135.0473; |
| 5 | trans-caffeoylgluconic acid-d4 | C_15_H_18_O_10_ | [M-H]^-^ | 358.0899 | 357.0854 | 3.0 | 195.0529;179.0363;135.0474;129.0216;99.0124;75.0136 |
| 6 | Chlorogenic acid | C_16_H_18_O_9_ | [M-H]^-^ | 354.0951 | 353.0902 | 3.7 | 191.0579 |
| 7 | Cryptochlorogenic acid | C_16_H_18_O_9_ | [M-H]^-^ | 354.0951 | 353.0904 | 4.1 | 191.0577;179.0365;135.0474; |
| 8 | trans-caffeoyl gluconate-methyl ester | C_16_H_20_O_10_ | [M-H]^-^ | 372.1420 | 371.0982 | 5.4 | 339.0731;179.0369;161.0265;135.0477;133.0323 |
| 9 | Caffeic acid | C_9_H_8_O_4_ | [M-H]^-^ | 180.0422 | 179.0373 | 0.1 | 135.0476 |
| 10 | Rutin | C_27_H_30_O_16_ | [M-H]^-^ | 610.1534 | 609.1513 | 1.9 | 301.0371;271.0258 |
| 11 | Hyperoside | C_15_H_10_O_7_ | [M+H] ^+^ | 302.0426 | 303.0509 | 2.5 | 285.0405;257.0451;229.0499;153.0187 |
| 12 | **Liquiritin** | C_21_H_22_O_9_ | [M-H]^-^ | 418.1176 | 417.1202 | 5.3 | 441.1268 [M＋Na]^+^, 419.136 8 [M+H]^+^, 257 [M+H-Glucose]^+^ |
| 13 | Isorhamnetin-3-O-rutinoside | C_29_H_34_O_17_ | [M+H] ^+^ | 654.1796 | 655.1879 | 1.5 | 509.1307;347.0778 |
| 14 | Dehydroevodiamine | C_19_H_15_N_3_O | [M+H] ^+^ | 301.1215 | 302.1295 | 2.2 | 286.0955;272.0807;258.1010;167.0591 |
| 15 | Quercetin | C_15_H_10_O_7_ | [M+H] ^+^ | 302.0426 | 303.0509 | 2.5 | 285.0405;257.0451;229.0499;153.0187 |
| 16 | **Glycyrrhizic acid** | C_42_H_62_O_16_ | [M+H] ^+^ | 822.4059 | 823.4057 | 6.3 | 823.4057 [M+H] ^+^, 647[M+H-Glucuronide acid] ^+^, 471 [M+H]^-^ |
| 17 | Limonin | C_26_H_30_O_8_ | [M+H] ^+^ | 470.1941 | 471.2029 | 3.4 | 425.1978;367.1918;161.0603 |
| 18 | Evodiamine | C_19_H_17_N_3_O | [M+H] ^+^ | 303.1372 | 304.1459 | 4.7 | 171.0911;161.0705;144.0804;134.0602;116.0497;106.0654 |
| 19 | Rutaecarpine | C_18_H_13_N_3_O | [M+H] ^+^ | 287.1059 | 288.1144 | 4.2 | 273.0883;244.0852;169.0749;145.0391 |
| 20 | **Glycyrrhetinic acid** | C_30_H_46_O_4_ | [M+H] ^+^ | 470.6843 | 471.7122 | 4.1 | 317.5223;427.5024 |
| 21 | Evocarpine | C_23_H_33_NO | [M+H] ^+^ | 339.2562 | 340.2591 | 1.0 | 298.2143;256.1674;242.1518;214.1213;200.1049;186.0893;  173.0818; 159.0667 |
| 22 | 1-methyl-2- (6Z,9Z)-10-penta-decadinenyl-4(1H)-quinolone | C_25_H_35_NO | [M+H] ^+^ | 365.2719 | 366.2809 | 4.7 | 256.1697;212.1071;186.0906;173.0827 |
| 23 | Dihydroevocarpine | C_23_H_35_NO | [M+H] ^+^ | 341.2719 | 342.2810 | 2.8 | 242.1544;228.1389;186.0904;173.0824 |

The identification was completed according to our previous work, and more information including instruments and conditions can be accessed (Li, 2020. Doi: 10.27180/d.cnki.gjxzc.2019.000017; Dong, 2019. Doi: 10.27180/d.cnki.gjxzc.2019.000238). The identification process was outlined below. ①, the compound database of EF and licorice was established by referring to the literatures and sorting out the compounds in this genus. ②, XIC Manager in Peakview software (Version1.2, AB Sciex) was used to analyze the Q-TOF/MS spectrum. Q-TOF/MS provides a variety of ion monitoring modes, such as parent ion scanning, daughter ion scanning, etc., which is conducive to the rapid detection of molecular ions. According to the retention time and its corresponding high resolution mass spectrometry (HRMS) of molecular ion, the molecular weight of the compound was determined. ③, according to the secondary fragments, reference material information, and relevant literature data, the fragmentation rule was found. ④, the retention time, the precise molecular weight, fragment ion, and literature data were integrated to determine the structure of the compound.
