# Supplementary material for: Hepatotoxicity Comparison of Crude and Licorice-Processed Euodiae Fructus in Rats With Stomach Excess-Cold Syndrome
Source: Front Pharmacol. 2021 Nov 23;12:756276. doi: 10.3389/fphar.2021.756276 (PMC8650065; doi:10.3389/fphar.2021.756276)
Supplement: Supplementary file 2 [file Table2.docx]

**Table S2 |** Comparison of general behaviors of rats on the 15^th^ day after oral administration (mean ± SD, n= 10).

| **Group** | | | **Food intake (g·d^-1^)** | **Water intake (mL·d^-1^)** | **Feces** **(0-12 h)** | | **Urine (0-12 h)** | |
| --- | --- | --- | --- | --- | --- | --- | --- | --- |
| **Drug** | **Extract** | **Dose（g·kg^-1^）** |  |  | **Weight (g)** | **Feature** | **Volume (mL)** | **Color** |
| Control | / | / | 24.4±1.5 | 23.5±2.2 | 9.2±0.6 | dry, granular | 14.2±1.2**^◇^** | faint yellow |
| Model | / | / | 23.3±1.8 | 22.7±3.1 | 8.8±0.5 | Loose, shapeless | 13.8±1.1**^◇◇^** | faint yellow |
| APAP | / | 0.21 | 16.0±2.1****** | 31.6±2.4***** | 6.2±0.4 | very dry, small granular | 18.6±1.4 | very dark yellow |
| CEF | WE | 1.05 | 22.6±1.9**^◇^** | 21.9±1.8**^◇◇^** | 9.1±0.8**^◇◇^** | dry, granular | 13.4±1.0**^◇◇^** | faint yellow |
|  |  | 5.25 | 20.7±1.3***^◇^** | 25.4±2.2**^◇^** | 7.8±0.6**^◇◇^** | dark brown, dry, small granular | 15.8±0.6**^◇^** | dark yellow |
|  |  | 10.5 | 17.1±1.4****** | 29.7±2.5***** | 6.7±0.6***** | black, dry, small granular | 18.1±1.1***** | very dark yellow |
|  | EE | 1.05 | 23.0±1.6**^◇◇^** | 22.6±1.8**^◇◇^** | 8.6±0.4**^◇◇^** | dry, granular | 13.5±0.5**^◇◇^** | faint yellow |
|  |  | 5.25 | 21.3±1.2***^◇^** | 24.1±1.4**^◇◇^** | 7.9±0.5**^◇◇^** | brown, dry, granular | 15.1±1.3**^◇^** | dark yellow |
|  |  | 10.5 | 18.4±1.3****** | 28.6±1.9***** | 7.0±0.3***^◇^** | black, dry, small granular | 17.4±1.2***** | very dark yellow |
|  | VO | 1.05 | 22.9±2.0**^◇^** | 23.0±2.1**^◇◇^** | 9.0±0.7**^◇◇^** | dry, granular | 14.3±0.7**^◇◇^** | faint yellow |
|  |  | 5.25 | 21.8±1.5**^◇^** | 26.5±1.4**^◇^** | 8.4±0.7**^◇◇^** | dry, small granular | 15.7±0.8**^◇^** | yellow |
|  |  | 10.5 | 19.4±2.3****** | 28.1±1.6***** | 7.4±0.6***^◇^** | faint brown, very dry, very small granular | 16.5±1.3***** | dark yellow |
| LPEF | WE | 1.05 | 23.2±1.5**^◇◇^** | 22.9±1.4**^◇◇^** | 8.8±0.8**^◇◇^** | dry, granular | 13.4±0.7**^◇◇^** | faint yellow |
|  |  | 5.25 | 21.3±2.5**^◇^** | 24.2±1.7**^◇^** | 8.4±0.7**^◇◇^** | dark brown, dry, small granular | 14.6±0.6**^◇^** | yellow |
|  |  | 10.5 | 18.9±1.7**^#^*** | 27.3±1.1***^#^** | 7.8±0.6***^#◇^** | black, dry, small granular | 17.0±0.7***^#^** | dark yellow |
|  | EE | 1.05 | 23.5±2.3**^◇◇^** | 23.3±1.6**^◇◇^** | 9.1±0.5**^◇◇^** | dry, granular | 13.9±0.8**^◇◇^** | faint yellow |
|  |  | 5.25 | 21.9±1.7**^◇^** | 25.8±1.1**^◇^** | 8.7±0.7**^◇◇^** | brown, dry, small granular | 14.4±0.9**^◇^** | yellow |
|  |  | 10.5 | 19.7±1.9***^#◇^** | 27.4±1.7***^#^** | 7.6±0.6***^#◇^** | dark brown, dry, small granular | 16.7±0.5***^#^** | dark yellow |
|  | VO | 1.05 | 23.3±1.6**^◇◇^** | 22.3±1.5**^◇◇^** | 9.1±0.8**^◇◇^** | dry, granular | 12.9±0.6**^◇◇^** | faint yellow |
|  |  | 5.25 | 22.6±2.1**^◇^** | 24.4±1.8**^◇^** | 8.5±0.7**^◇◇^** | dry, small granular | 14.5±0.8**^◇^** | yellow |
|  |  | 10.5 | 21.3±1.0***^#◇^** | 25.9±1.5**^#◇^** | 7.9±0.8***^#◇^** | brown, dry, very small granular | 15.6±1.1***^#◇^** | dark yellow |

Values are mean ± SD of ten replicated samples; *vs* control group, *p* < 0.05 (*****) and *p* < 0.01 (******); *vs* CEF, *p* < 0.05 (**^#^**) and *p* < 0.01 (**^##^**); *vs* APAP, *p* < 0.05 (**^◇^**) and *p* < 0.01 (**^◇◇^**).
