# Supplementary material for: Hepatotoxicity Comparison of Crude and Licorice-Processed Euodiae Fructus in Rats With Stomach Excess-Cold Syndrome
Source: Front Pharmacol. 2021 Nov 23;12:756276. doi: 10.3389/fphar.2021.756276 (PMC8650065; doi:10.3389/fphar.2021.756276)
Supplement: Supplementary file 3 [file Table5.docx]

**Table S5.** Comparison of different extracts on serum biochemical indexes in rats with stomach excess-cold syndrome.

| **Group** | | | **ALT (IU·L^-1^)** | **AST (IU·L^-1^)** | **TNF-α (μg·L^-1^)** | **IL-1β (μg·L^-1^)** | **IL-6 (μg·L^-1^)** |
| --- | --- | --- | --- | --- | --- | --- | --- |
| **Drug** | **Extract** | **Dose (g·kg^-1^)** |  |  |  |  |  |
| Control | / | / | 34.23±2.53 | 64.41±5.57 | 107.2±7.8 | 43.37±4.19 | 33.83±3.13 |
| Model | / | / | 36.51±2.41 | 72.09±6.93 | 97.31±8.93 | 55.88±6.05 | 34.36±3.81 |
| APAP | / | 0.21 | 54.34±4.93****** | 128.4±13.3****** | 235.5±19.2****** | 124.7±10.9****** | 45.19±4.28****** |
| CEF | WE | 1.05 | 39.71±3.27**^◇◇^** | 99.27±8.65**^◇◇^** | 137.6±17.9**^◇◇^** | 70.37±7.12**^◇◇^** | 37.86±3.08**^◇◇^** |
|  |  | 5.25 | 45.63±4.45***^◇^** | 125.5±12.2****^◇^** | 214.6±20.6****^◇^** | 117.4±10.6****^◇^** | 41.28±3.55***^◇^** |
|  |  | 10.5 | 59.25±4.98****** | 145.2±14.7****** | 240.0±26.8****** | 138.8±12.9****** | 48.82±4.76****** |
|  | EE | 1.05 | 39.02±4.21**^◇◇^** | 82.32±8.12**^◇◇^** | 132.5±13.4**^◇◇^** | 66.34±7.06**^◇◇^** | 36.50±3.86**^◇◇^** |
|  |  | 5.25 | 42.24±4.19***^◇◇^** | 115.6±10.7****^◇^** | 145.6±14.1****^◇◇^** | 87.11±7.59****^◇◇^** | 40.85±4.04***^◇^** |
|  |  | 10.5 | 49.08±5.09****** | 129.2±13.8****** | 170.5±16.9****^◇^** | 102.6±10.2****^◇^** | 44.37±4.48****** |
|  | VO | 1.05 | 38.09±3.42**^◇◇^** | 88.17±8.47**^◇◇^** | 128.5±12.1**^◇◇^** | 64.39±6.16**^◇◇^** | 35.24±3.62**^◇◇^** |
|  |  | 5.25 | 41.03±4.03***^◇◇^** | 103.8±9.6****^◇^** | 147.3±13.9****^◇◇^** | 85.05±7.89***^◇◇^** | 39.39±4.06***^◇^** |
|  |  | 10.5 | 51.07±4.91****** | 123.5±13.4****** | 162.4±15.1****^◇◇^** | 98.5±11.02****^◇^** | 42.33±4.48***^◇^** |
| LPEF | WE | 1.05 | 37.24±2.08**^◇◇^** | 84.53±9.26**^◇◇^** | 114.7±10.2**^◇◇^** | 61.66±6.09**^◇◇^** | 33.64±3.19**^◇◇^** |
|  |  | 5.25 | 41.11±3.91*****^#^**^◇◇^** | 93.91±8.99******^#^**^◇◇^** | 177.1±19.4******^#^**^◇^** | 109.5±9.8******^##^**^◇^** | 38.14±3.27*****^#^**^◇^** |
|  |  | 10.5 | 47.36±4.53******^#^**^◇^** | 105.0±11.4******^##^**^◇◇^** | 212.4±24.9******^##^**^◇^** | 103.5±11.1******^##^**^◇^** | 41.85±4.06******^##^**^◇^** |
|  | EE | 1.05 | 36.31±3.74**^◇◇^** | 74.71±7.00**^◇◇^** | 117.3±10.8**^◇◇^** | 59.74±5.41**^◇◇^** | 34.16±2.93**^◇◇^** |
|  |  | 5.25 | 40.18±3.83*****^#^**^◇◇^** | 92.84±9.17******^##^**^◇◇^** | 121.4±12.1******^#^**^◇◇^** | 79.92±5.81******^##^**^◇◇^** | 36.32±3.59^#^**^◇^** |
|  |  | 10.5 | 44.22±4.36******^#^ | 107.5±12.1******^##^**^◇^** | 155.5±13.3******^##^**^◇^** | 87.64±6.85******^##^**^◇◇^** | 40.77±4.17*****^#^**^◇^** |
|  | VO | 1.05 | 36.13±3.36**^◇◇^** | 76.34±8.05**^◇◇^** | 108.9±9.9**^◇◇^** | 51.18±4.00**^◇◇^** | 33.97±2.84**^◇^** |
|  |  | 5.25 | 38.43±3.29*****^#^**^◇◇^** | 89.21±8.77******^##^**^◇◇^** | 119.6±12.5******^#^**^◇◇^** | 71.36±7.05******^##^**^◇◇^** | 36.65±3.57*****^#^**^◇^** |
|  |  | 10.5 | 47.14±3.82******^#^ | 101.8±11.5******^##^**^◇^** | 139.6±13.1******^##^**^◇◇^** | 84.32±8.59******^##^**^◇◇^** | 39.67±3.81*****^#^**^◇^** |

Values are mean ± SD of ten replicated samples; *vs* control group, *p* < 0.05 (*****) and *p* < 0.01 (******); *vs* CEF, *p* < 0.05 (**^#^**) and *p* < 0.01 (**^##^**); *vs* APAP, *p* < 0.05 (**^◇^**) and *p* < 0.01 (**^◇◇^**).
