# Supplementary material for: Hepatotoxicity Comparison of Crude and Licorice-Processed Euodiae Fructus in Rats With Stomach Excess-Cold Syndrome
Source: Front Pharmacol. 2021 Nov 23;12:756276. doi: 10.3389/fphar.2021.756276 (PMC8650065; doi:10.3389/fphar.2021.756276)
Supplement: Supplementary file 4 [file Table3.docx]

**Table S3.** Comparison of different extracts on the hepatic mitochondria Na^+^, K^+^-ATPase and Ca^2+^-Mg^2+^-ATPase activity in rats with stomach excess-cold syndrome.

| **Group** | | | **Na^+^, K^+^-ATPase (μmolPi·mgprot^-1^·h^-1^)** | **Ca^2+^-Mg^2+^-ATPase (μmolPi·mgprot^-1^·h^-1^)** |
| --- | --- | --- | --- | --- |
| **Drug** | **Extract** | **Dose (g·kg^-1^)** |  |  |
| Control | / | / | 1.182±0.133 | 1.574±0.123 |
| Model | / | / | 1.105±0.101 | 1.411±0.112 |
| APAP | / | 0.21 | 0.588±0.065****** | 0.675±0.084****** |
| CEF | WE | 1.05 | 0.901±0.126**^◇◇^** | 1.269±0.121**^◇◇^** |
|  |  | 5.25 | 0.708±0.107***^◇^** | 0.841±0.166****^◇^** |
|  |  | 10.5 | 0.497±0.076****** | 0.605±0.053****^◇◇^** |
|  | EE | 1.05 | 0.925±0.108**^◇◇^** | 1.208±0.116**^◇◇^** |
|  |  | 5.25 | 0.833±0.061***^◇^** | 0.873±0.114****^◇^** |
|  |  | 10.5 | 0.591±0.063****** | 0.694±0.084****** |
|  | VO | 1.05 | 1.006±0.149**^◇◇^** | 1.264±0.116**^◇◇^** |
|  |  | 5.25 | 0.867±0.096***^◇^** | 0.906±0.106****^◇^** |
|  |  | 10.5 | 0.641±0.105****** | 0.713±0.116****** |
| LPEF | WE | 1.05 | 1.018±0.138**^◇◇^** | 1.311±0.135**^◇◇^** |
|  |  | 5.25 | 0.813±0.065*****^#^**^◇◇^** | 0.937±0.179*****^##^**^◇◇^** |
|  |  | 10.5 | 0.623±0.041******^##^ | 0.724±0.062******^##^**^◇^** |
|  | EE | 1.05 | 1.083±0.114**^◇◇^** | 1.413±0.131**^◇◇^** |
|  |  | 5.25 | 0.912±0.105*****^#^**^◇◇^** | 0.985±0.147******^#^**^◇◇^** |
|  |  | 10.5 | 0.657±0.076******^##^**^◇^** | 0.762±0.106******^##^**^◇^** |
|  | VO | 1.05 | 1.188±0.105^#^**^◇◇^** | 1.457±0.158**^◇◇^** |
|  |  | 5.25 | 0.929±0.109*****^#^**^◇◇^** | 1.099±0.148******^#^**^◇◇^** |
|  |  | 10.5 | 0.778±0.113******^##^**^◇^** | 0.874±0.127******^##^**^◇^** |

Values are mean ± SD of ten replicated samples; *vs* control group, *p* < 0.05 (*****) and *p* < 0.01 (******); *vs* CEF, *p* < 0.05 (**^#^**) and *p* < 0.01 (**^##^**); *vs* APAP, *p* < 0.05 (**^◇^**) and *p* < 0.01 (**^◇◇^**).
