# Supplementary material for: Hepatotoxicity Comparison of Crude and Licorice-Processed Euodiae Fructus in Rats With Stomach Excess-Cold Syndrome
Source: Front Pharmacol. 2021 Nov 23;12:756276. doi: 10.3389/fphar.2021.756276 (PMC8650065; doi:10.3389/fphar.2021.756276)
Supplement: Supplementary file 5 [file Table4.docx]

**Table S4.** Comparison of different extracts on the serum SOD and MDA activity in rats with stomach excess-cold syndrome.

| **Group** | | | **SOD (U·mgprot^-1^)** | **MDA (nmol·mgprot^-1^)** | **The ratio of**  **SOD to MDA** |
| --- | --- | --- | --- | --- | --- |
| **Drug** | **Extract** | **Dose (g·kg^-1^)** |  |  |  |
| Control | / | / | 252.8±23.3 | 2.174±0.287 | 116.3±8.5 |
| Model | / | / | 236.8±21.9 | 2.258±0.302 | 104.9±11.2 |
| APAP | / | 0.21 | 142.5±18.8****** | 8.468±0.655****** | 16.68±1.24 |
| CEF | WE | 1.05 | 201.8±22.6**^◇◇^** | 2.833±0.296**^◇◇^** | 71.26±6.69**^◇◇^** |
|  |  | 5.25 | 167.5±21.5***^◇^** | 5.139±0.314****^◇◇^** | 32.46±3.65****^◇◇^** |
|  |  | 10.5 | 93.4±10.6****^◇◇^** | 10.12±1.07****^◇^** | 9.244±0.657****^◇^** |
|  | EE | 1.05 | 209.7±20.2**^◇◇^** | 2.729±0.231**^◇◇^** | 76.86±6.73**^◇◇^** |
|  |  | 5.25 | 175.6±18.9***^◇^** | 4.783±0.296****^◇◇^** | 36.73±2.15****^◇◇^** |
|  |  | 10.5 | 114.1±17.5****^◇^** | 8.953±0.755****** | 12.67±1.42***^◇^** |
|  | VO | 1.05 | 216.5±22.5**^◇◇^** | 2.477±0.259**^◇◇^** | 92.72±8.36**^◇◇^** |
|  |  | 5.25 | 205.6±20.4***^◇^** | 4.291±0.275***^◇◇^** | 49.67±4.52****^◇◇^** |
|  |  | 10.5 | 155.9±16.1***** | 6.091±0.582****^◇^** | 25.63±1.98***^◇^** |
| LPEF | WE | 1.05 | 217.4±24.1**^◇◇^** | 2.292±0.265**^◇◇^** | 94.92±9.19**^◇◇^** |
|  |  | 5.25 | 183.3±22.4*****^#^**^◇◇^** | 4.277±0.275******^#^**^◇◇^** | 42.89±3.77******^#^**^◇◇^** |
|  |  | 10.5 | 122.8±12.2******^##^**^◇^** | 9.331±1.005******^##^**^◇^** | 14.27±1.23****** |
|  | EE | 1.05 | 218.3±21.6**^◇◇^** | 2.353±0.217**^◇◇^** | 92.83±8.15**^◇◇^** |
|  |  | 5.25 | 188.5±21.7*****^#^**^◇^** | 3.894±0.331******^#^**^◇◇^** | 48.41±4.52******^##^**^◇◇^** |
|  |  | 10.5 | 127.5±18.3******^##^**^◇^** | 7.011±0.713******^##^**^◇^** | 17.75±1.33******^#^ |
|  | VO | 1.05 | 229.1±21.1**^◇◇^** | 2.338±0.209**^◇◇^** | 100.1±11.4**^◇◇^** |
|  |  | 5.25 | 213.4±21.8*****^#^**^◇^** | 3.935±0.311******^#^**^◇◇^** | 73.64±6.62******^##^**^◇◇^** |
|  |  | 10.5 | 171.6±18.1******^##^**^◇◇^** | 5.173±0.458******^##^**^◇◇^** | 36.34±2.58******^##^**^◇◇^** |

Values are mean ± SD of ten replicated samples; *vs* control group, *p* < 0.05(*****) and *p* < 0.01(******); *vs* CEF, *p* < 0.05(**^#^**) and *p* < 0.01(**^##^**); *vs* APAP, *p* < 0.05(**^◇^**) and *p* < 0.01(**^◇◇^**).
