# Supplementary material for: Hepatotoxicity Comparison of Crude and Licorice-Processed Euodiae Fructus in Rats With Stomach Excess-Cold Syndrome
Source: Front Pharmacol. 2021 Nov 23;12:756276. doi: 10.3389/fphar.2021.756276 (PMC8650065; doi:10.3389/fphar.2021.756276)
Supplement: Supplementary file 6 [file Table6.docx]

**Table S6.** Comparison of different extracts on apoptosis associated protein expressions in rats with stomach excess-cold syndrome.

| **Group** | | | **Bax** | **Bcl-2** | **Caspase 3** |
| --- | --- | --- | --- | --- | --- |
| **Drug** | **Extract** | **Dose**  **(g·kg^-1^)** |  |  |  |
| Control | / | / | 1 | 1 | 1 |
| Model | / | / | 1.232±0.121 | 0.917±0.181 | 1.151±0.073 |
| APAP | / | 0.21 | 3.165±0.249 | 0.575±0.049 | 4.984±0.382 |
| CEF | WE | 1.05 | 1.628±0.226**^◇◇^** | 0.698±0.068**^◇◇^** | 1.604±0.142**^◇◇^** |
|  |  | 5.25 | 2.628±0.247***^◇^** | 0.603±0.058***** | 4.523±0.316***^◇^** |
|  |  | 10.5 | 3.375±0.315****** | 0.437±0.092****^◇^** | 7.056±0.485****^◇◇^** |
|  | EE | 1.05 | 1.451±0.108**^◇◇^** | 0.791±0.094**^◇◇^** | 1.348±0.137**^◇◇^** |
|  |  | 5.25 | 2.417±0.153****^◇◇^** | 0.682±0.085***^◇^** | 3.083± 0.136****^◇◇^** |
|  |  | 10.5 | 3.184±0.222****** | 0.549±0.055****** | 5.415±0.493****** |
|  | VO | 1.05 | 1.429±0.078**^◇◇^** | 0.823±0.112**^◇◇^** | 1.253±0.172**^◇◇^** |
|  |  | 5.25 | 2.247±0.154****^◇◇^** | 0.761±0.095***^◇^** | 2.479±0.231****^◇◇^** |
|  |  | 10.5 | 2.939±0.223****** | 0.608±0.063****** | 4.856±0.403****** |
| LPEF | WE | 1.05 | 1.313±0.105**^◇◇^** | 0.793±0.103**^◇◇^** | 1.427±0.117**^◇◇^** |
|  |  | 5.25 | 2.074±0.191*****^#^**^◇◇^** | 0.717±0.101*****^#^**^◇^** | 3.629±0.246******^##^**^◇◇^** |
|  |  | 10.5 | 2.851±0.253******^##^ | 0.653±0.056******^##^ | 5.381±0.533******^##^ |
|  | EE | 1.05 | 1.283±0.116**^◇◇^** | 0.835±0.112**^◇◇^** | 1.186±0.094**^◇◇^** |
|  |  | 5.25 | 1.98±0.189*****^#^**^◇◇^** | 0.741±0.095*****^#^**^◇^** | 2.716± 0.146******^#^**^◇◇^** |
|  |  | 10.5 | 2.467±0.187******^##^**^◇◇^** | 0.604±0.089******^##^ | 4.817±0.245******^##^**^◇^** |
|  | VO | 1.05 | 1.314±0.231**^◇◇^** | 0.902±0.123**^◇◇^** | 1.073±0.118**^◇◇^** |
|  |  | 5.25 | 1.793±0.182*****^#^**^◇◇^** | 0.813±0.108*****^#^**^◇^** | 2.061±0.165******^#^**^◇◇^** |
|  |  | 10.5 | 2.239±0.164******^##^**^◇◇^** | 0.754±0.084******^##^ | 4.257±0.321******^##^**^◇^** |

Values are mean ± SD of three replicated samples; *vs* control group, *p* < 0.05 (*****) and *p* < 0.01 (******); *vs* CEF, *p* < 0.05 (**^#^**) and *p* < 0.01 (**^##^**); *vs* APAP, *p* < 0.05 (**^◇^**) and *p* < 0.01 (**^◇◇^**).
