# Supplementary material for: Hepatotoxicity Comparison of Crude and Licorice-Processed Euodiae Fructus in Rats With Stomach Excess-Cold Syndrome
Source: Front Pharmacol. 2021 Nov 23;12:756276. doi: 10.3389/fphar.2021.756276 (PMC8650065; doi:10.3389/fphar.2021.756276)
Supplement: Supplementary file 7 [file Image1.pdf]

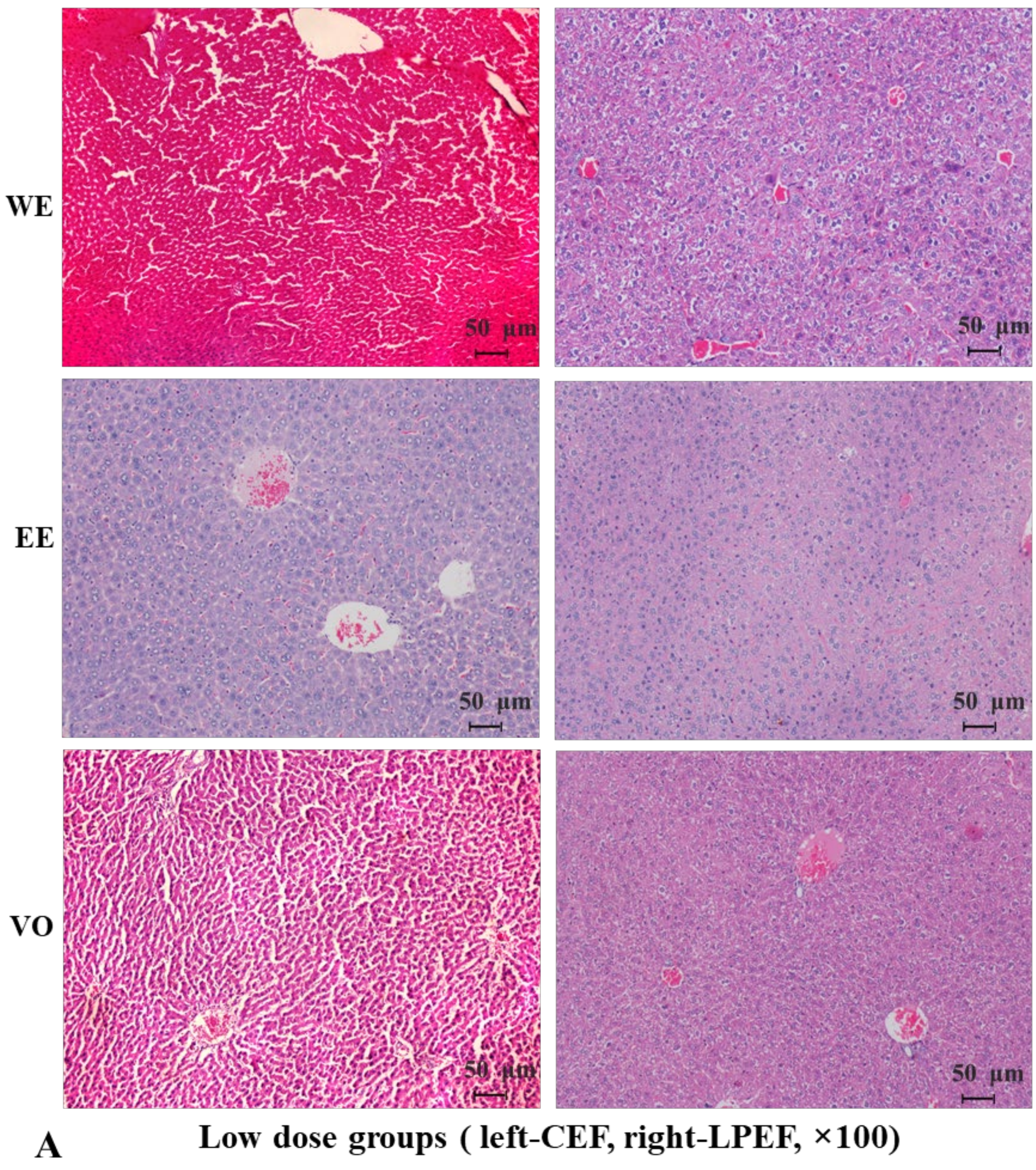


Low dose groups ( left-CEF, right-LPEF, ×100)

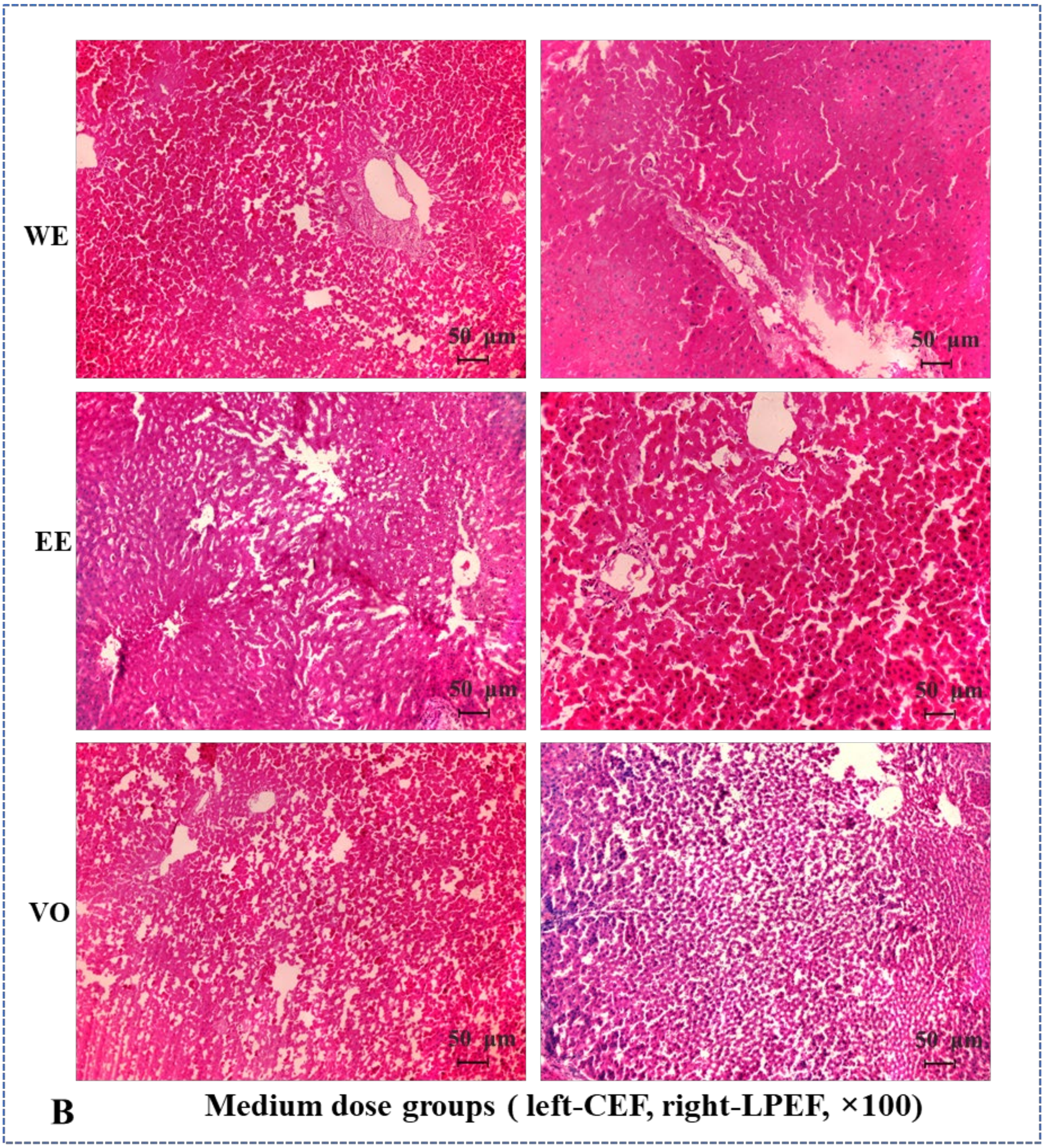


**Fig S1 |** Effects of CEF and LPEF on pathomorphology of liver issue in rats with stomach excess-cold syndrome treated with different extracts. A, Low dose group; B, Medium dose group.
